# Group membership dictates the neural correlates of social optimism biases

**DOI:** 10.1038/s41598-020-58121-4

**Published:** 2020-01-24

**Authors:** Mihai Dricu, Laurent Schüpbach, Mirko Bristle, Roland Wiest, Dominik A. Moser, Tatjana Aue

**Affiliations:** 1 University of Bern, Bern, Switzerland; 2 Institute for Diagnostic and Interventional Neuroradiology, Inselspital Hospital, Bern, Switzerland

**Keywords:** Cognitive neuroscience, Social behaviour, Social neuroscience, Human behaviour

## Abstract

Optimism bias, i.e. expecting the future to hold more desirable than undesirable outcomes, also extends to people that we like or admire. However, it remains unknown how the brain generates this social optimism bias. In this study, respondents estimated the likelihood of future desirable and undesirable outcomes for an in-group and three out-groups: warm-incompetent, cold-competent, and cold-incompetent. We found a strong social optimism bias for the in-group and the warm out-group and an inverted pattern for the cold-incompetent out-group. For all groups, scores of social optimism bias correlated with the brain activity in structures that respondents differentially engaged depending on the target social group. In line with our hypotheses, evaluating the in-group recruited the ventromedial prefrontal cortex and the precuneus/posterior cingulate cortex, whereas evaluating the warm out-group engaged the posterior insula, mid cingulate cortex, and somatosensory cortices. These findings suggest different underlying cognitive mechanisms of social optimism bias for these groups, despite similar behavioural patterns. Thinking about the cold out-groups recruited the right anterior temporal lobe, and temporoparietal junction. Evaluating the cold-incompetent out-group additionally recruited the anterior insula, inferior frontal cortex and dorsomedial frontal cortex. We discuss these neuroimaging findings with respect to their putative cognitive functions.

## Introduction

Human minds are highly capable of solving problems at hand, anticipating prospective issues and benefits, and planning accordingly. At the core of these skills lies the capacity to gauge the likelihood of future events^1–3^. The way we assess the likelihood of future events in the general population is different from how we assess it for ourselves^4–6^, for those close to us^7–10^, and for in-group^11,12^ and out-group members^11–19^. Through various motivational^20,21^ and cognitive mechanisms^22,23^, we manifest an optimism bias whenever we think about our future and the future of those close to us^4,8,10,11,13^ but not of acquaintances or dissimilar others^8,11,14,24,25^. Specifically, we expect that the future holds significantly more desirable than undesirable outcomes for ourselves and those we identify with^26^. Despite a modest body of behavioural research on social optimism bias, i.e. optimism manifested towards people that we like and feel close to, very little is known about how the brain gives rise to this phenomenon^18^. The primary focus of the current study was to investigate the neural correlates of group membership-driven optimism biases. To pursue this research aim and inform our hypotheses, we considered the neuroimaging literature on person perception and social cognition.

A hallmark of social cognition is that individuals think differently about in-group and out-group members^27–32^. Although what determines in-group and out-group membership is flexible and highly contextual^30,33^, people perceive in-group and out-group members as part of different entities with respect to one or more attributes^32^. Unsurprisingly, deliberating over in-group members is associated with a brain network that is involved in self-referential processing^32,34–40^: the ventromedial prefrontal cortex extending into the anterior cingulate cortex (vmPFC/ACC) and the precuneus extending into the posterior cingulate cortex (PCUN/PCC). Deliberating over out-groups typically engages brain structures associated with accessing conceptual knowledge and stereotypical thinking^41–44^: the temporoparietal junction^45–48^ (TPJ), the anterior temporal lobes^49–51^ (ATL), the dorsomedial frontal cortex^52,53^ (dmFC) and potentially the inferior frontal cortex^54^ (IFC).

A formal theoretical model of social cognition is the Stereotype Content Model (SCM), which states that we think and feel about others in terms of two orthogonal dimensions, *perceived warmth*, i.e. how (un)likeable someone is, and *perceived competence*, i.e. how (un)respectable someone is^55–62^. The SCM predicts that the warmth dimension weighs more in impression formation than the competence dimension^56,60,63^. According to the SCM, out-groups can be placed on a continuum by virtue of their combined warmth and competence dimensions^60,64–66^. For example, there can be “mild”, warm but not competent out-groups (e.g. the elderly and individuals with disabilities^67,68^), “moderate”, cold but competent out-groups (e.g. successful business people^58,64,69^) and “extreme”, cold and not competent out-groups (e.g. drug and substance abusers^60^). Typical social emotions associated with each type of out-group encapsulate pity and sympathy for the warm-incompetent out-group, envy and jealousy for the cold-competent out-group and contempt and disgust for the cold-incompetent out-group^58,62^.

The tenet of the SCM is that there are more out-groups that trigger ambivalent emotions and prejudice than clear-cut disparaging or threatening reactions^58,70^. It is important to note that SCM does not contradict previous research on the role of threat induced by out-groups; rather, it opens the concept of out-group to incorporate other non-traditional forms^56^. Because of the combination of the warmth and competence dimensions into four groups of traits, some groups share one or the other dimension. A few neuroimaging studies of SCM have suggested that highly-praised in-groups, and mild and moderate out-groups engage similar neural structures by virtue of their shared traits of either high warmth or high competence, respectively^71,72^. For example, the vmPFC was equally recruited when evaluating the warm and competent in-group, as well as two out-groups with which it shared one of the SCM dimensions: a warm but not competent out-group, and a cold but competent out-group. The authors suggested that this is indicative of similar cognitive mechanisms of evaluation on account of the shared dimensions^71^. By contrast, evaluating an extreme out-group (cold and not competent) was associated with insular and amygdala activity in a pattern characteristic of dehumanized perception^71^. These findings are somewhat in contrast with the wider literature on social neuroscience which has found qualitative differences in the perception of in-groups and out-groups, with the latter invariably engaging regions of stereotypical thinking (e.g.^32,37,38,73^). A secondary goal of the present study was, therefore, to properly address the apparent disagreement between the SCM predictions and the social neuroscience findings, at large.

Our current study adapted the paradigm developed by Dricu *et al*.^74^ for functional magnetic resonance imaging (fMRI). In short, participants rated the likelihood of a set of identical desirable and undesirable target events occurring to four different fictional characters. These characters mapped onto each of the four quadrants of the SCM: a warm and competent in-group, a warm and not competent out-group, a cold and competent out-group, and a cold and not competent out-group. An earlier behavioural study based on this paradigm showed that respondents manifested a prominent desirability bias towards the in-group and the warm out-group, i.e. more desirable events were forecasted than undesirable events^74^. Respondents did not manifest a desirability bias towards the cold and competent out-group (i.e. desirable and undesirable events would occur equally often) but they expected significantly more undesirable than desirable events to happen for the “extreme” cold and not competent out-group. In other words, the direction of the desirability bias was inversed for the latter group. These findings, along with the evidence from the neuroimaging literature on social groups, have informed our following hypotheses.

## Hypotheses

Our main guiding hypothesis was that the phenomenon of desirability bias is generated in the brain structures that the respondents differentially engage depending on a target’s group membership. Based on the wider social cognition literature, we postulated that the biases towards different social groups are driven by different cognitive processes. Specifically, we hypothesized that the desirability bias manifested for the in-group member can be traced to regions of self-referential processing, as informed by the general social cognition literature (**H1**), e.g. vmPFC/ACC and PCUN/PCC^34–40,75^. We note that the SCM predicts that similar cognitive processes are recruited whenever we evaluate social groups that share a dimension^60^. This would transcend group membership, i.e. in-group vs. out-group, and the shared SCM dimension would be enough to generate these similarities. Yet, only a single neuroimaging study has found evidence for this SCM prediction^71^, and this finding has not been replicated^72^. In line with the extant neuroimaging literature on social groups, therefore, we postulated that responses to the in-group generally differ from the ones to different kinds of out-groups.

Moreover, regarding out-group members, we predicted that not all out-groups are perceived homogenously^64,67,76^. For example, the associated emotions and behavioural tendencies of the mild (warm and not competent) out-group are conducive to empathy (even more so than the in-group^66,77^), whereas the emotions and behavioural tendencies of the moderate and extreme (i.e., cold) out-groups are not^66,77–82^. In fact, if given the chance, people are more likely to attack moderate out-group members (active harm^66^, e.g. apply painful but not deadly electric shocks) and sacrifice extreme out-group members (passive harm^83^, e.g. respondents only have to let events follow their natural course in a Trolley moral dilemma) than mild out-group members. These findings strongly suggest that the warm out-group is unique among all out-groups^66,67,70,84^. We thus expected that the desirability bias would manifest for a warm (and not competent) out-group target and could be traced to regions associated with compassion and empathic concern (**H2**), such as the posterior insula^85–87^, the somatosensory cortices^85–91^, the cingulate cortex^87,89^, and potentially the motor cortices^92,93^. The desirability bias for the cold out-groups, on the other hand, would be associated with the brain activity in a network of regions associated with stereotypical thinking (**H3**), e.g. the TPJ^45–48^, ATL^49–51^, dmFC^52,53^, and, possibly, the IFC^54^.

Finally, we generated a hypothesis about the unique pattern of the desirability bias for the “extreme” out-group members, i.e. cold and not competent. Previously, participants expected members of this out-group to experience significantly more undesirable than desirable events^74^. That is, the desirability bias manifested towards this out-group was inversed compared to the other social groups. Past research has found that members of such extreme out-groups are often dehumanized^94^, on account of the SCM predicting that extreme out-group members trigger emotions of contempt and disgust^62^. Therefore, we predicted that the desirability bias for the alcoholic character can be traced to the anterior insula^71,72^, the amygdala^71,72^ and, possibly, the dmFC^95,96^ (**H4**), regions associated with feelings of disgust and repulsion.

## Results

### Behavioural results

#### Likelihood estimates

We computed four one-sample t-tests (one for each character) on the differences in z-standardized likelihood estimates for desirable events and undesirable events. Participants expressed a desirability bias for all four fictional characters, i.e. desirable events were rated significantly different from undesirable events (all Ps < 0.0005, corrected for multiple testing).

**Figure 1:**
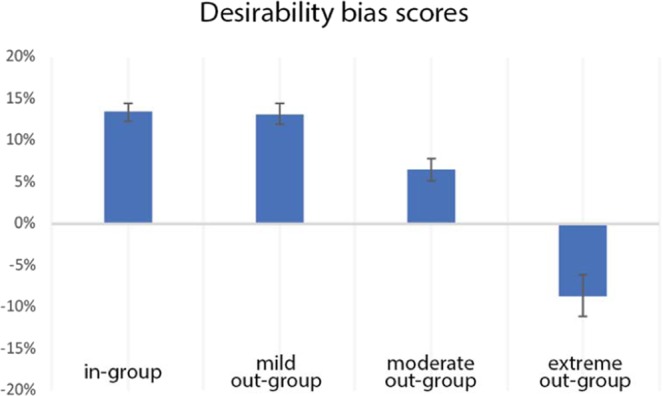
Desirability bias scores for the four social groups, calculated as the difference between the likelihood estimates for desirable events and undesirable events. The student character served as the warm-competent in-group member, the elderly as the mild (warm-incompetent) out-group, the businessperson as the moderate (cold-competent) out-group, and the alcoholic as the extreme (cold-incompetent) out-group.

A 2 (high/low warmth) × 2 (high/low competence) repeated measures ANOVA was computed on the differences in z-standardized likelihood estimates for desirable and undesirable events to investigate any differences between characters on the magnitude and direction of desirability bias. We found main effects of warmth (F (1, 44) = 78.84, p < 0.0005, η_p_^2^ = 0.627) and competence (F (1, 44) = 10.88, p = 0.002, η_p_^2^ = 0.188), with the latter being qualified by an interaction effect (F (1, 44) = 29.72, p < 0.0005, η_p_^2^ = 0.387). In effect, participants expressed a significantly higher desirability bias for warm characters than for cold characters (Diff = 13.94%, SD = 11.36%). Follow-up tests revealed that the in-group (M = 12.61%, MSE = 1.15%, SD = 7.48%) and the warm out-group (M = 13.68%, MSE = 1.63%, SD = 10.92%) generated similar magnitude levels of the desirability bias (t (44) = −0.560, p = 0.578, Cohen’s d = −0.08). Within cold characters, however, the direction of the desirability bias was dependent on the target’s level of competence: the extreme out-group was expected to experience significantly more undesirable events than desirable ones, whereas it was the reverse for the moderate out-group (Fig. 1). Regarding the magnitude of the bias, the less competent cold out-group members elicited a significantly higher bias (M = -7.58%, MSE = 2.58%, SD = 17.33%) than did the competent cold out-group (M = 5.53%, MSE = 2.58%, SD = 9.09%; t (44) = 5.020, p < 0.0005, Cohen’s d = 0.76).

#### Reaction times

To determine whether there are any differences in reaction times, we computed a warmth (cold vs. warm) × competence (high vs. low) repeated measures analysis of variance on deliberation times, i.e. the last release of the button press minus the first press of the button, separately for desirable and undesirable events. For desirable events, we found a marginally significant main effect of competence (F (1, 44) = 4.32, p = 0.044, ηp^2^ = 0.089) but no main effect of warmth (F (1, 44) = 1.77, p = 0.284, ηp^2^ = 0.026) or an interaction effect between warmth and competence (F (1, 44) = 0.072, p = 0.407, ηp^2^ = 0.016). Competent characters were rated faster (M = 2690.26 ms, MSE = 136.78 ms) than incompetent characters (M = 2825.49 ms, MSE = 124.29 ms). For undesirable events, there were no main effect of warmth (F (1, 44) = 0.02, p = 0.888, ηp^2^ < 0.001), of competence (F (1, 44) = 1.80, p = 0.186, ηp^2^ = 0.039) or an interaction between warmth and competence (F (1, 44) = 0.369, p = 0.546, ηp^2^ = 0.008).

### Functional imagining data

#### Hypothesis H1: desirability bias for the in-group

**Figure 2:**
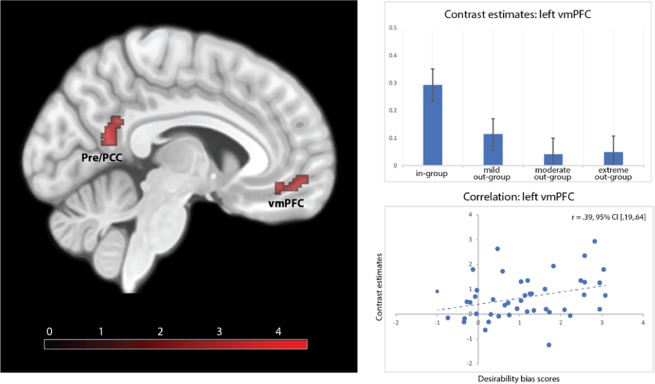
Three-way conjunction analysis of the contrasts in-group vs. mild out-group, in-group vs. moderate out-group, and in-group vs. extreme out-group. The left-side image depicts brain activation in precuneus extending into the posterior cingulate cortex (Pre/PCC) and in the ventromedial prefrontal cortex (vmPFC). The right-side top image shows the contrast estimates separately for each social group in the left vmPFC. The right-side bottom image shows the brain-behaviour correlation between contrast estimates in the left vmPFC and the desirability bias scores for the in-group. p < 0.001 at voxel-level and p < 0.01 at cluster-level.

To compare responses to the in-group with the responses to the three different out-groups, the following three contrasts were computed at the first level and subsequently taken to the second level where a conjunction analysis was performed: in-group vs. mild out-group, in-group vs. moderate out-group and in-group vs. extreme out-group. The conjunction analysis revealed significant activation in the PCUN extending into the PCC and in the vmPFC (Fig. 2, Table 1).

**Table 1:** Regions that are significantly more active when evaluating the in-group member vs. the out-group members, as shown by the three-way conjunction analysis of the contrasts in-group vs. mild out-group, in-group vs. moderate out-group, and in-group vs. extreme out-group. p < 0.001 and a cluster-level p < 0.01.

Region	Cluster size	z-score	MNI coordinates
Precuneus/posterior cingulate cortex	81	4.34	−4, −58, 16
Ventromedial prefrontal cortex	76	3.94	−8, 52, −8
	^	3.71	−6, 40, −12

^part of the same functional cluster.

To directly test our hypothesis H1 **(**desirability bias manifested toward the in-group member is associated with regions of self-referential processing), we correlated the desirability bias for the in-group member (compared to out-group members) with the mean parameter estimates of the clusters identified in the PCUN/PCC and vmPFC from the three-way conjunction analysis. In line with H1 we found a significant correlation (Benjamini-Hochberg corrected for two tests) between the desirability bias for the in-group and brain activation in the vmPFC (r = 0.39, 95% CI [0.19, 0.64]; Spearman rho = 0.34, p = 0.021). The correlation with the parameter estimates in the PCUN/PCC did not reach statistical significance (r = 0.27, 95% CI [−0.01, 0.50]; Spearman rho = 0.30, p = 0.045, Benjamini-Hochberg corrected for two tests; Supplementary Fig. S1).

#### Hypothesis H2: desirability bias for warm out-group

A similar approach was taken to investigate the characteristics of the three out-groups, i.e. hypotheses H2, H3, and H4. The following three contrasts were computed at the first level and then taken to the second level: mild out-group vs. in-group, moderate out-group vs in-group and extreme out-group vs. in-group. Subsequently, a one-way flexible factorial model (factor: minuend character) with three levels (mild out-group, moderate out-group, extreme out-group) was computed on the three second-level contrasts. We used the outcome of this single SPM matrix to investigate the characteristics of the out-groups and address the three hypotheses. To determine which brain regions, if any, were similarly activated by the warm out-group and the two cold out-groups with respect to the in-group, we performed a three-way conjunction analysis on these second-level images. This analysis did not reveal any significant overlap in brain activation with respect to the in-group.

**Figure 3:**
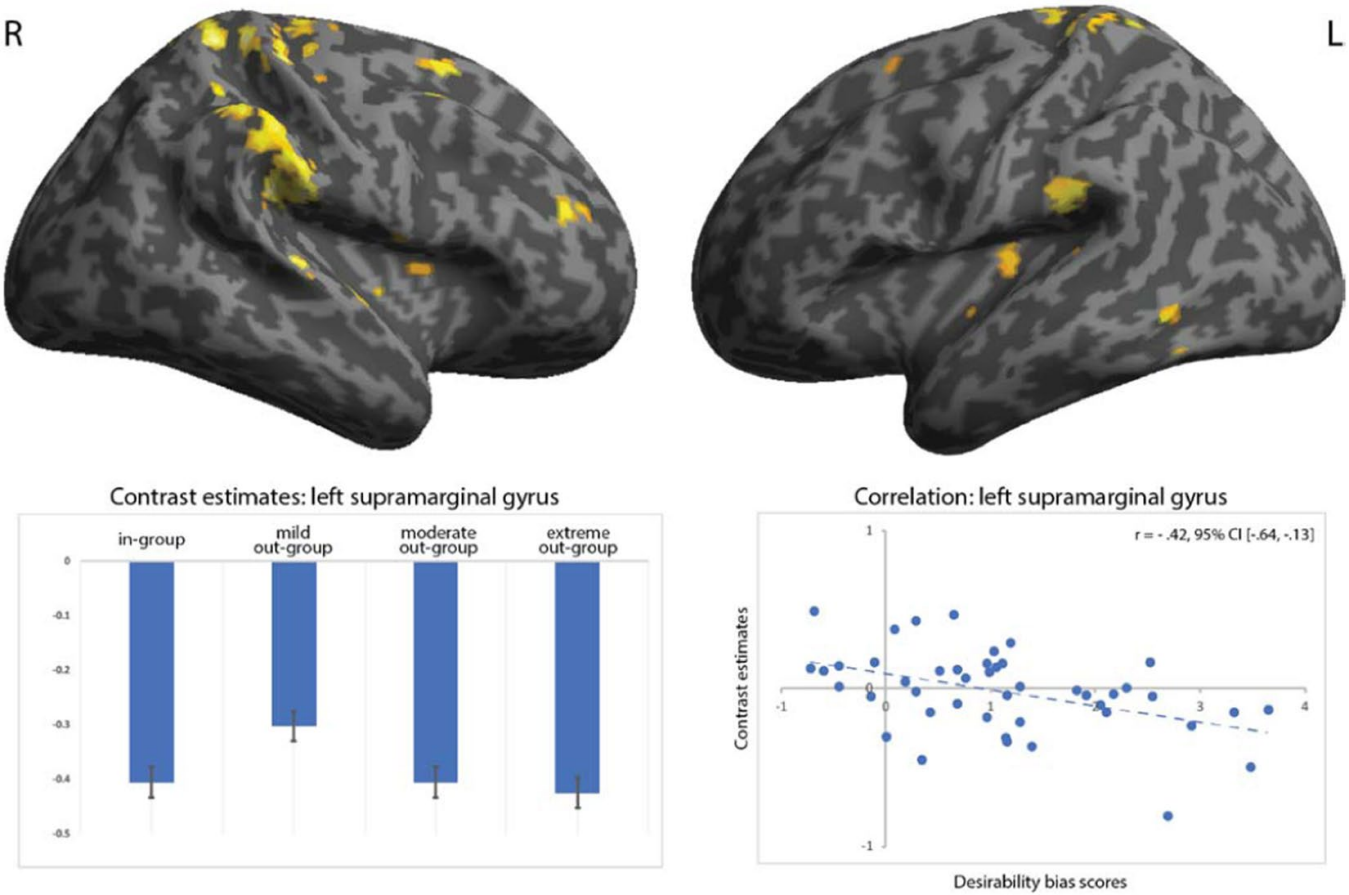
Top two images depict right-side (R) and left-side (L) brain activation from the flexible factorial contrast 2 × (mild out-group − in-group) − [(moderate out-group − in-group) + (extreme out-group − in-group)]. For the labelling of these regions, please consult Supplementary Table S1. The bottom two images show parameter estimates for the four social groups in the left supramarginal gyrus (bottom left-side) and brain-behavioural correlation between the respective desirability bias for the mild out-group and contrast estimates in the left supramarginal gyrus (bottom right-side). p < 0.001 at voxel-level and p < 0.01 at cluster-level.

In preparation for testing hypothesis H2, i.e. desirability bias for the warm out-group being associated with regions of empathic concern, we used the previously described factorial model to perform a pairwise comparison between the warm out-group and the cold out-groups. This allowed to determine whether any brain regions are uniquely recruited by the warm out-group compared to the cold out-groups. Extensive activation was found in regions associated with empathy and compassion for others, i.e. bilateral posterior insula, the cingulate cortex, and secondary and primary somatosensory cortices (Fig. 3, Supplementary Table S1). We then performed brain-behaviour correlations between the desirability bias for the warm out-group and the contrast estimates found in our seven hypothesized regions, i.e. the cingulate cortex, bilateral insula, and bilateral primary and secondary somatosensory cortices. Significant correlations (Benjamini-Hochberg corrected for seven tests) were found between the desirability bias for the warm out-group (compared to the in-group and cold out-groups) and the brain activity in the left supramarginal gyrus/secondary somatosensory cortex (r = −0.42, 95% CI [−0.64, −0.13]; Spearman rho = −0.45, p = 0.002). No significant brain-behaviour correlations were found for the remaining six regions (left posterior insula (r = −0.27, 95% CI [−0.53, 0.05]; Spearman rho = −0.19, p = 0.217), right posterior insula (r = 0.15, 95% CI [−0.14, 0.41]; Spearman rho = 0.15, p = 0.340), mid cingulate cortex (r = −0.14, 95% CI [−0.40, 0.15]; Spearman rho = −0.14, p = 0.378), left primary somatosensory (r = −0.17, 95% CI [−0.46, 0.16]; Spearman rho = −0.20, p = 0.196), right primary somatosensory cortex (r = −0.21, 95% CI [−0.51, 0.13]; Spearman rho = −0.19, p = 0.211) and right supramarginal/secondary somatosensory cortex (r = −0.17, 95% CI [−0.43, 0.13]; Spearman rho = −0.22, p = 0.152, Supplementary Fig. S1).

#### Hypothesis H3: desirability bias for cold out-groups

**Figure 4:**
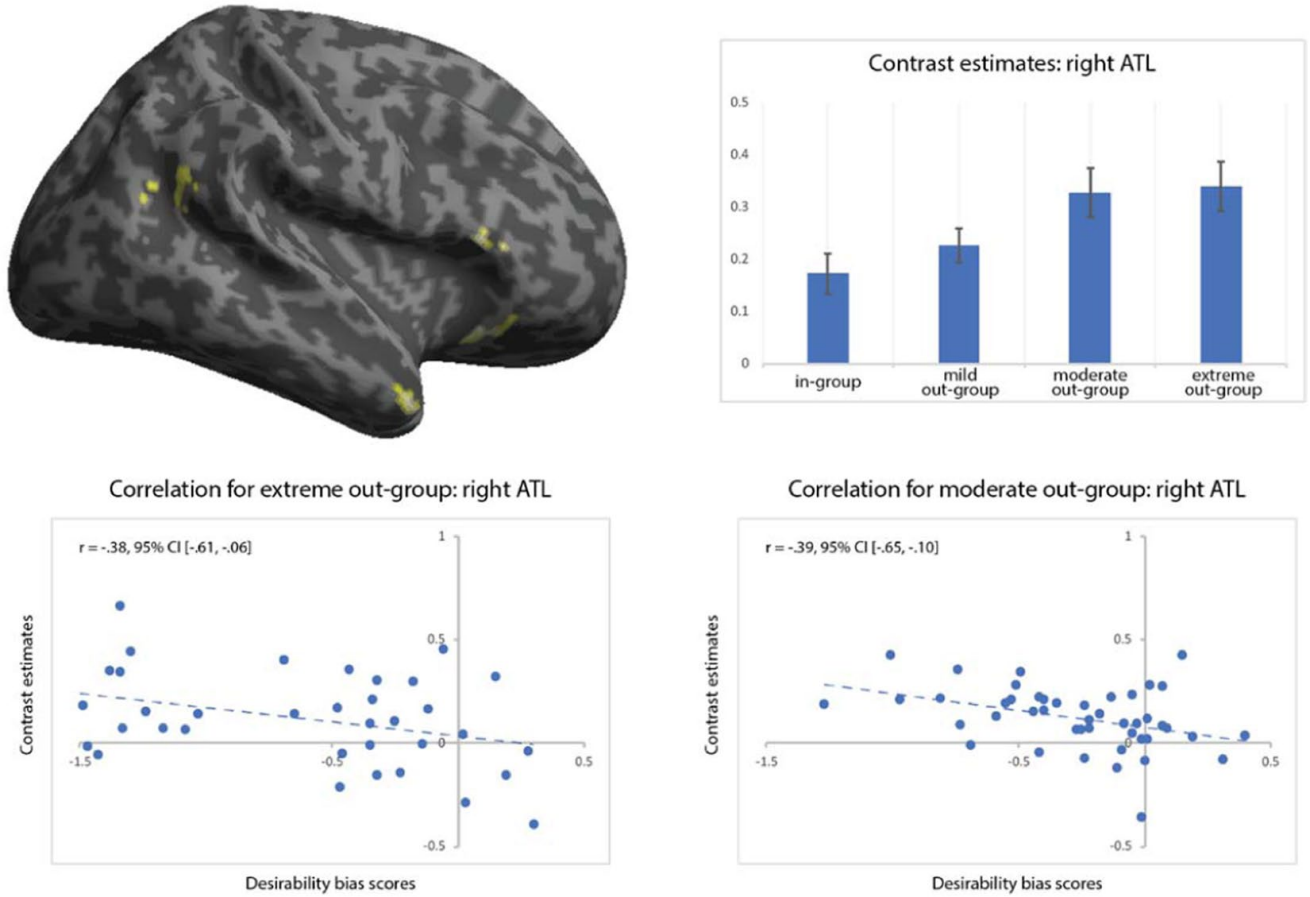
The top left image depicts brain activation from the two-way conjunction analysis between the contrasts [moderate out-group – in-group] and [extreme out-group – in-group]. For the labelling of these regions, please consult Supplementary Table 2. Top right image shows contrast estimates for the four social groups in the right anterior temporal lobe (ATL). The bottom two images show correlation analyses between contrast estimates in the right ATL and desirability bias scores for the extreme out-group (compared to in-group; bottom left side) and the moderate out-group (compared to in-group; bottom right-side), respectively. p < 0.001 at voxel-level and p < 0.01 at cluster-level.

To prepare for testing hypothesis **H3**, i.e. desirability bias for the cold out-group members is associated with the BOLD signal in regions of stereotypical thinking, we performed two analyses. First, a two-way conjunction analysis on the two second-level contrast images moderate outgroup vs. in-group and extreme out-group vs. in-group revealed a significant overlap in brain activation in the IFC, the ATL and the TPJ (Fig. 4, Table 2). Second, a [−2 1 1] contrast was calculated in the above (see analyses related to H2) specified one-way flexible factorial analysis to determine whether any brain regions are uniquely recruited by the cold out-groups compared to the warm out-group (and the in-group). Calculation of this contrast revealed extensive activation in regions such as anterior temporal pole, dmFC, and superior temporal sulcus (Supplementary Table S2).

**Table 2:** Regions that are significantly more active when evaluating the cold out-groups vs. the in-group, as shown by the two-way conjunction analysis of the contrasts moderate (cold-competent) out-group vs. in-group and extreme (cold-incompetent) out-group vs. in-group. p < 0.001 and a cluster-level p < 0.01.

Region	Cluster size	z-score	MNI coordinates
Pars triangularis (inferior frontal cortex)	126	4.88	58, 20, 6
	^	3.80	56, 32, 8
Anterior temporal lobe	101	4.43	48, 16, −38
Pars orbitalis (inferior frontal cortex)	85	4.32	38, 24, −16
Temporoparietal junction	121	3.87	44, −56, 22
	^	3.75	52, −54, 24
	^	3.49	64, −50, 20

^part of the same functional cluster as above.

To directly address hypothesis **H3**, we correlated the desirability biases for our two cold out-groups, on one hand, and the parameter estimates found in the four hypothesized regions, i.e. the ATL, the TPJ, and the pars triangularis and pars orbitalis of the IFC. Two significant correlations (Benjamini-Hochberg corrected for eight tests) were found between the desirability bias for moderate out-group (compared to in-group) and the brain activity in the ATL (r = −0.39, 95% CI [−0.65, −0.097]; Spearman rho = −0.37, p = 0.012) and the pars orbitalis of the IFC (r = −0.34, 95% CI [−0.62, −0.04]; Spearman rho = −0.39, p = 0.009). Parameter estimates for the TPJ (r = −0.19, 95% CI [−0.49, 0.02]; Spearman rho = −0.26, p = 0.082) and the pars triangularis of the IFC (r = −0.26, 95% CI [−0.53, 0.03]; Spearman rho = −0.24, p = 0.120) did not reach statistical difference (Benjamini-Hochberg corrected for eight tests; Supplementary Fig. S2). Furthermore, a significant correlation (Benjamini-Hochberg corrected for eight tests) was found between the desirability bias for the extreme out-group (compared to in-group) and the brain activity in the ATL (r = −0.38, 95% CI [−0.61, −0.06]; Spearman rho = −0.38, p = 0.011) but not in the TPJ (r = −0.16, 95% CI [−0.43, 0.19]; Spearman rho = −0.16, p = 0.294), the pars triangularis (r = −0.22, 95% CI [−0.50, 0.09]; Spearman rho = −0.22, p = 0.149) or the pars orbitalis of the IFC (r = −0.21, 95% [−0.48, 0.11]; Spearman rho = 0.21, p = 0.173; Supplementary Fig. S2).

#### Hypothesis H4: desirability bias for the extreme out-group

**Figure 5:**
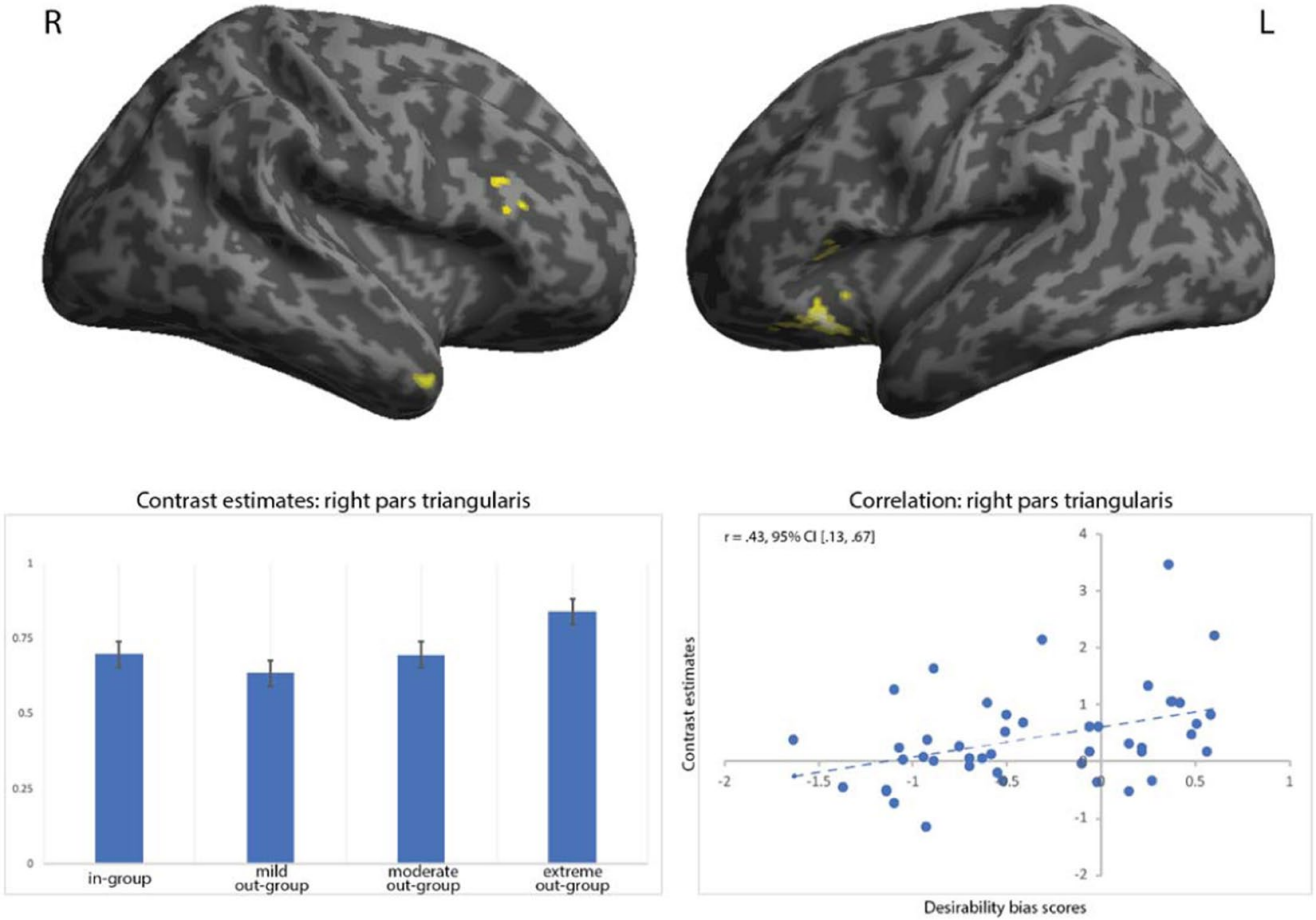
Top two images depict brain activation from the flexible factorial contrast 2 × (extreme out-group − in-group) − [(moderate out-group − in-group) + (mild out-group − in-group)]. For labelling of the regions, please consult Supplementary Table 3. Bottom left-side image illustrates contrast estimates for the four social groups. Bottom right-side image illustrates brain-behaviour correlation analysis between contrast estimates in the right pars triangularis and the desirability bias scores for the extreme out-group (compared to the other groups). p < 0.001 at voxel-level and p < 0.01 at cluster-level.

Lastly, to determine whether the desirability bias manifested towards the extreme out-group correlates with regions of dehumanized perception (**H4**), we first used the previous one-way flexible factorial model to contrast the cold, extreme out-group against the cold, moderate out-group and the warm out-group (all against the in-group as baseline). In line with our hypothesis, this analysis revealed activation in the left pars orbitalis of the IFC extending into the anterior insula, bilateral dmFC, the right ATL and two clusters in the right pars triangularis of the IFC (Fig. 5, Table 3). A statistically significant correlation (Benjamini-Hochberg corrected for six tests) was found between the desirability bias for extreme out-group (compared to the other social groups) and the brain activity in the right pars triangularis of IFC (r = 0.43, 95% CI [0.13, 0.67]; Spearman rho = 0.45, p = 0.002). No significant correlations (Benjamini-Hochberg corrected for six tests) were found for the left pars orbitalis (r = −0.19, 95% CI [−0.44, 0.12], Spearman rho = −0.09, p = 0.557), the right dmFC (r = −0.09, 95% CI [−0.39, 0.21], Spearman rho = −0.04, p = 0.809), the left dmFC (r = 0.05, 95% CI [−0.23, 0.32], Spearman rho = 0.12, p = 0.434), the ATL (r = 0.04, 95% CI [−0.27, 0.33], Spearman rho = 0.02, p = 0.910) or the second cluster in pars triangularis (r = 0.21, 95% CI [−0.07, 0.47], Spearman rho = 0.21, p = 0.165; Supplementary Fig. S3).

**Table 3:** Regions that are significantly more active when evaluating the extreme (cold-incompetent) out-group than the mild (warm-incompetent) out-group and the moderate (cold-competent) out-group, with the in-group as baseline. p < 0.001 and a cluster-level p < 0.01.

Region	Cluster size	z-score	MNI coordinates
Pars orbitalis and anterior insula	388	5.15	−30, 18, −16
	^	4.45	−32, 22, 2
	^	4.25	−38, 18, −12
Dorsomedial frontal cortex	112	4.2	6, 52, 30
	^	3.76	6, 48, 38
	^	3.1	2, 54, 22
Dorsomedial frontal cortex	34	3.68	−6, 52, 26
Pars triangularis	25	3.63	46, 24, 22
Anterior temporal lobe	31	3.53	52, 8, −38
Pars triangularis (inferior frontal cortex)	28	3.49	58, 30, 16
	^	3.26	50, 32, 14

^part of the same functional cluster as above.

For the interested reader, we also computed the same analyses described above separately for desirable and undesirable events (Supplementary Tables S3 and S4), as well as a 2 × 4 (valence × character) ANOVA (Supplementary Table S5). Supplementary Fig. S4 provides parameter estimates for some of the regions found significant in the main effect of character and the interaction effect of character and valence. These analyses provide further details on the social cognition about the in-group versus out-groups in desirable and undesirable situations.

## Discussion

Our study aimed to investigate the neural correlates of social optimism bias depending on a target’s social group membership. These neural signatures can inform about the cognitive mechanisms underlying different forms of social optimism bias. The literature has shown that people evaluate in-group and out-group members fundamentally different^27^. Through various motivational and cognitive mechanisms, we perceive the in-group more favourably than the out-group^27,30,97,98^. For example, maintaining a high self-esteem is a strong motivational factor to praise in-group members^28,29^, while the lack of first-hand knowledge about certain categories of people means that stereotypes will be used to infer conclusions about them^41,99^. This also manifests in the way we think about the future: we anticipate that desirable events will happen significantly more often than undesirable events for people that we like, whereas the opposite pattern is expected for those we dislike^74^. In line with the SCM, and fully replicating our behavioural data in a previous study^74^, we show that participants manifested a strong desirability bias, i.e. expected more desirable than undesirable events for the in-group and the warm (but not competent) out-group members. A desirability bias was also present for the cold and competent out-group, likely due to its perceived levels of skill and expertise, but its magnitude was significantly lower than for the in-group and warm out-group (and it had not been significant in our earlier study). Together, these findings support the SCM prediction that the warmth dimension has a stronger impact on impression formation about out-groups (thereby making the bias for the warm out-group similar to the one for the in-group than the competence dimension). Lastly, participants expected significantly more undesirable than desirable events to happen to the cold-incompetent out-group.

Behaviourally, our respondents manifested the same magnitude and direction of the optimism bias for the in-group and the warm out-group. Our neuroimaging findings strongly suggest that this similarity is likely an epiphenomenon rather than the outcome of similar cognitive mechanisms generated by the shared warmth dimension. Instead, the two biases are likely regulated by different cognitive mechanisms. Evaluating in-group members increased neural activity in the vmPFC and the PCUN extending into the PCC, both regions supporting self-referential processing^34–40,75^. Self-referential processing refers to the class of cognitive processes in which the individual distinguishes stimuli related to one’s own self from those that are not relevant to one’s own concerns^75^. This includes evaluating oneself on personality traits and accessing autobiographical memories. Although lesion studies and non-invasive brain stimulation would provide stronger support for the causal role of the vmPFC and PCUN/PCC in self-referential processing, medial brain structures are difficult to study with these methods. Neurosynth is a viable alternative to these methods and offers a formal measure of the degree of confidence in the reverse inference, by estimating posterior probabilities, i.e. the degree to which a region of interest is *selectively* activated by the cognitive process of interest^100,101^. A formal search for our peak coordinates in the PCUN/PCC and vmPFC on the Neurosynth platform (www.neurosynth.org) revealed that the top three highest posterior probabilities for the PCUN/PCC were 89% that it indexes “autobiographical memory”, followed by a probability of 79% that it indexes “episodic memory”, and 77% “self-referential processing”. The top three highest posterior probabilities for our peak coordinate in the vmPFC were 85% for “autobiographical memory”, followed by 83% for “remembering” and 82% for default-network. Together, these considerations yield strong support to our interpretation that the network of vmPFC and PCUN/PCC might indeed reflect self-referential processes. Of these regions, the brain activity in the vmPFC significantly correlated with the behavioural scores for desirability bias manifested towards the in-group characters. In light of this finding, it is likely that respondents recognize the in-group members as relevant to oneself and evaluate them on the basis of their personal experiences which are then extrapolated (e.g. self-anchoring^102^) and/or based on direct interaction with in-group members (i.e. first-hand knowledge of their idiosyncratic behaviour). In turn, the strong desirability bias manifested for the in-group might stem from a similar self-serving bias^40^ that underlies personal optimism^103^. In other words, respondents may be driven by a motivation to reach and maintain positive self-esteem via in-group favouritism^104^.

Although similar in its behavioural pattern to the in-group, the desirability bias manifested towards the warm out-group might come from a different mechanism, namely an empathic concern for their well-being^82,105^. Largely consistent with our a priori hypothesis, when participants evaluated the warm (and incompetent) out-group, they engaged a network of brain regions consisting of bilateral insula, the mid cingulate cortex, and bilateral primary and secondary somatosensory cortices. We argue that the significance of this network may indeed relate to an empathic response of respondents towards the warm out-group. In support for such an interpretation of the data, the same regions have been implicated in two separate neuroimaging meta-analyses in the empathy for others in pain based either on facial expression alone^106^ or inferred based on the perception of acute pain infliction to body parts^107^ (i.e. perceiving needles pricking the hand). These regions also coincide with the results of an independent meta-analysis on the effects of loving-kindness meditation on the brain^108^. Loving-kindness is a type of mediation that fosters compassion and deep genuine sympathy for those stricken by misfortune, together with an earnest wish to ease this suffering^109^. Of the regions revealing characteristic activation for the mild out-group, the right supramarginal gyrus (i.e. secondary somatosensory cortex) could be successfully linked with the magnitude of the desirability bias. That empathy could be the mechanism underlying the desirability bias for the mild out-group receives further support from the demonstration of causal links between the supramarginal gyrus and empathic responses in studies of transcranial magnetic stimulation (primary somatosensory cortex^110,111^, the right supramarginal gyrus^91,112,113^, motor cortex^92,114^), and transcranial direct current stimulation (right supramarginal gyrus^115–119^ and motor cortex^93^).

We note that, although the current results suggest that the desirability bias towards the warm out-group may stem from empathic concern and compassion, future research should formally investigate this by manipulating the degree of compassion or empathy. Furthermore, one may ask why members of a warm but incompetent out-group may trigger emotions of pity and sympathy^60,66,70^. Although out-groups are, generally, sources of threat and vigilance^120^, warm out-groups represent a special exception^66,77–82^. This out-group’s lack of competence puts them at a higher risk for undesirable outcomes yet they cannot be blamed for their fate on account of their warmth traits^67,70^. Consequently, respondents may overestimate the likelihood of positive outcomes and/or underestimate the likelihood of negative ones for warm out-groups to reduce the cognitive dissonance, perhaps as a result of just-world beliefs^121,122^.

In contrast, evaluating the two cold out-groups was associated with activity in a network of right-side brain regions, i.e. the TPJ, ATL, and the IFC. In our hypotheses, we had argued that respondents might rely on stereotypical thinking about these groups to inform their likelihood estimates^41–44^. Stereotypical thinking consists of several stages, including encoding and storing conceptual associations between a social group behavioural or mental attributes, accessing these associations from semantic memory and matching them to perceived social group in a task-dependent manner^44^. Several authors argue that at the heart of this matching process is the self-other differentiation^48^, in which the right TPJ likely plays a crucial role, as shown by studies on transcranial magnetic stimulation^123–129^, direct current stimulation^130^ and multivariate pattern analysis^131,132^. Although it is still debated how the exact mechanism of self-other differentiation occurs^133^, it is a necessary mechanism in social cognition and it describes the ability to distinguish between representations of our own actions, perceptions and emotions, and those of others, and toggle between the two perspectives^45–48^. Although the right TPJ has a much broader role in visual attention and flexibly switching between interoceptive and exteroceptive perception^134^, one hypothesis is that evolution may have re-purposed voluntary attention and spatial orientation for social cognition^135^.

Bilateral ATL plays play a crucial role in encoding and storing both social and non-social conceptual knowledge^49,50^, as shown by studies on lesion patients^136–143^, transcranial magnetic stimulation^136,144–154^, intracranial recordings^155^ and metabolic dysfunctions^156,157^. Our findings show that the behavioural scores of desirability bias for the businessperson and the alcoholic significantly correlate with the brain activity in the right ATL. Evaluating the alcoholic additionally recruited the anterior insula, the dmPFC and other patches in the ATL and IFC (pars triangularis). Because the anterior insula has been causally linked to the experience of disgust in studies of invasive^158–160^ and non-invasive^161,162^ brain stimulation, and the SCM predicts disgust to be a unique reaction to cold-incompetent out-groups^62^, it would be tempting to suggest that our findings provide support for the latter. Our findings concerning the network of empathic concern towards the elderly characters also seem to support the SCM prediction for the social emotions that respondents might experience^62^.

Surprisingly, of the regions uniquely active to the alcoholic character, it was the brain activity in the right pars triangularis that significantly correlated with the corresponding desirability bias for the alcoholic. The right pars triangularis has been involved in a wide range of perceptual decision-making tasks^163–165^, i.e. deliberating over what is perceived based on sensory information^166^. Computationally, perceptual decision-making consists of matching the incoming perceptual evidence to a mental template that can take the form of episodic memories or semantic knowledge of how an object or a person looks like and behaves^167–169^. More than taking on a passive role in the matching process, the right pars triangularis is directly involved in the resolution of stimulus conflict, i.e. deciding which characteristics of a complex stimulus are relevant for accurately perceiving it^170^. Considering our current results and the previous research on the IFC within the framework of the SCM, one possible explanation for the pars triangularis’ involvement in the desirability bias for the extreme out-group rests on successfully resolving the conflict between the mental template of a negative stereotype for individuals characterized by low warmth and low competence and the incoming perceptual information (e.g. the character experiencing a desirable situation). Conflict resolution may then be achieved by attributing lower likelihood estimates to the desirable situations and/or higher estimates to the undesirable situations.

We would like to note the limitations of our study. While our sample size of 45 participants adheres to the minimum recommendation provided by Yarkoni and Braver for brain-behavioural correlations^171^, it is still far from an ideal size to uncover moderate effect sizes^172^. We tried to mitigate this logistical aspect with robust skipped correlations^173^, in line with the most recent recommendations for best practices in brain-behavioural correlations^174^. In addition, we had strong a priori hypotheses about our brain regions, we considered network activation and we corrected for multiple comparisons using the Benjamini-Hochberg formula whenever there was more than one hypothesized region. Overall, we are confident that our brain-behavioural correlations are not spurious. The present literature would benefit from replicating our current findings with a larger sample size, which should provide increased power to detect brain-behaviour correlations in more regions of our networks of interest^172^. For example, activity in only one region in the network of interest for hypothesis H2 was significantly correlated with the desirability bias scores but this does not necessarily mean it is the only region with this feature. We also note that the visual analogue scale that participants used to rate the likelihood estimates always displayed the value “0%” in the left and “100%” in the right, while the slider always started at “50%”. While the fixed labels for the extremes of the scale is unlikely to have had any confounding effects on our data^175^, the starting position of the slider may have influenced the differential rating of the characters. Future studies can investigate whether a randomly positioned slider would maintain our current results.

A general limitation in neuroimaging studies is the use of reverse inferences^100,101^, in which researchers are limited in their confidence of associating cognitive functions to brain activation. While this limitation cannot be fully avoided, we tried to increase the degree of confidence in our brain-function associations with several steps. First, we based our hypotheses in previous literature of social cognition, by focusing on networks of regions instead of isolated structures, and we only discuss findings that supported our predictions. Second, we provide references from multiple methods to support our brain-function associations, such as meta-analyses, large scale databases, lesion studies, and invasive and non-invasive brain stimulation studies. Third, whenever cross-referencing brain-function associations with multiple methods was not possible, we emphasized the speculative nature of these associations.

Overall, the present study’s findings build on and extend our previous behavioural results^74^ and suggest that the SCM can only be applied to predict behavioural, emotional and neural responses to out-groups but not in-groups. In contrast to the former, responses to the in-group may be influenced by reference to personal experience rather than stereotypes of warmth and competence. Such an interpretation can also explain the disagreement in predictions between SCM and the general social cognition literature concerning shared cognitive processes across dimensions (secondary goal of the current study). Although the in-group shares warmth traits with the warm out-group and competence traits with the cold-competent out-group at a conceptual level^60^, the neural responses do not back up these shared traits. Instead, in-group and out-group members are perceived and evaluated qualitatively different, above and beyond warmth and competence traits. However, warmth and competence traits may still hold considerable predictive power over the neural responses to out-groups. These findings could also spark interest in the field of social cognition and social neuroscience to move forward from studying obvious out-groups (e.g. ethnic or racial out-groups) to investigating a wider range of out-groups, in line with SCM predictions. At a practical level, our results could help inform policy makers and health promoters about the underpinnings of social optimism bias. For example, our inherent optimistic expectancies for the future of in-group and warm out-group members can have detrimental effects on taking steps to prevent accidents and illnesses. Risk communicators aware of the cognitive processes underlying these expectancies can make it particularly salient in their message that a lack of experience with a particular hazard is not protective against future experiences or that idle compassion is not sufficient to prevent threats. Conversely, our pessimistic expectancies for the cold-incompetent out-group can have detrimental effects on social policies, by questioning their efficacy and stalling their implementation. Understanding the cognitive mechanisms behind these expectancies can help policy makers fine tune campaigns to reduce prejudice against cold-incompetent out-groups.

In summary, we show that a specific form of social optimism bias, i.e. desirability bias, correlates with the brain activity in those structures that respondents differentially engage when assessing members of different social groups. Evaluating the in-group recruited regions that have been previously associated with self-referential processing, whereas evaluating the warm out-group engaged regions that have been repeatedly linked with compassion and empathic concern. Thinking about cold out-groups triggered activation in regions of stereotypical thinking. Future studies on desirability bias could benefit from incorporating other neuroimaging methods, such as multivariate pattern analysis and functional connectivity analysis. In our results, only a few of the regions inside our hypothesized networks of interest were correlated with the desirability bias. While a larger sample size might have picked up additional correlations^172^, this might also suggest that the complex phenomenon of the desirability bias extends beyond the network of interest (e.g. compassion and empathic concern) to other networks that are recruited in a task-based manner^176^ and that perhaps it may not be captured by contrast-based fMRI analyses^177^. Future studies using functional connectivity with the seed regions in the structures that we have identified here may prove a further step of inquiry. In the only other neuroimaging study on social optimism bias, the magnitude of the bias correlated with the degree of differential functional connectivity between the dorsal striatum and regions of visual perception and attention^18^.

An interesting avenue for future research would further consist of an extension of the SCM to out-groups that possess high-warmth, high-competence traits in order to examine whether it is the quadrant of the model that lacks prognostic value or whether the restricted predictive power of the model is limited to the in-group, above and beyond warmth and competence traits. To our knowledge, no other SCM study has done so. Furthermore, potential respondents in future studies could include in-group members of a different SCM quadrant, such as a high-warmth, low-competence social group (e.g. the elderly^67,178^) and compare the pattern of results with our student population. Finally, future research should incorporate ratings of warmth and competence, as well as other emotional descriptors (i.e. assessment of perceived level of threat) as moderators of the fMRI analysis. The SCM already incorporates emotional descriptions into its predictions^62^ but a formal investigation would be welcome.

## Methods

### Participants

Forty-eight Swiss university students participated in this study. They were recruited via fliers and the local participant pool at the University of Bern. In exchange for their participation, they received either course credits or monetary payment (25 CHF per hour). As inclusion criteria, participants had to be German-speaking full-time university students, aged between 18 and 40 years, right-handed, and have a Body Mass Index between 18.5 and 25. Self-reported neurological and psychiatric disorders, as well as MRI contraindications were exclusion criteria. Three participants were further excluded due to excessive movement or suspicious behavioural scores (i.e. the values “0%”, “50%” or “100%” represented more than three standard deviations above the percentages of estimates at the sample level). The analyses reported below are based on the final sample of forty-five participants (thirty-one females, *M* = 23.22, SD = 3.58). The communicated purpose of the study was gaining insight into the neural correlates of thinking about the future.

All participants gave informed and written consent for their participation. The local ethics committee of the University of Bern approved all experimental protocols and methods of data collection, data handling and analysis. All methods and experimental protocols were performed in accordance with the guidelines and regulations of the local Ethics Committee of the University of Bern, Switzerland.

### Paradigm

Data collection took place at the Insel University Hospital of Bern, Switzerland. Participants had to evaluate the likelihood estimates of four fictional characters experiencing an identical set of desirable and undesirable events, as described in Dricu *et al*.^74^. Sixteen desirable and sixteen undesirable events had been previously matched on valence (i.e. how far apart the event’s valence was from a hypothetical middle point, implying an emotionally neutral event), emotional impact, personal experience, perceived controllability, and frequency in the general population. The four characters reflected each of the quadrants of the two-dimensional space of warmth and competence^60,65^. A student character served as a high warmth, high competence in-group for our participants^11,27,28,74,104^. Three additional characters served as out-groups. An elderly person (high warmth, low competence^67^); served as a mild out-group. A successful businessperson (low warmth, high competence^58,64,69^); served as a moderate out-group. Finally, an alcoholic character served as an extreme out-group (low on both warmth and competence^60^).

The experiment was programmed with E-Prime 2.0 Professional (version 2.0.10.353; Psychology Software Tools, Pittsburgh, USA). The task was approximately thirty minutes long and was split in four blocks of equal lengths. During each block, the thirty-two unique scenarios were paired with each of the four characters in a Latin Square Design and presented in a randomized order. At the beginning of the first block, written instructions were displayed on the computer screen, asking participants to familiarize themselves with the four characters and informing them of the task, i.e. provide likelihood estimates for each of the four characters experiencing certain events (Supplementary Fig. S5). Each trial started with a jittered fixation cross (1.5s–3s), followed by a single screen which contained the target scenario (i.e. a single still animation of the target character involved in the target event), a one-sentence description of the target event displayed in the middle of the screen and a visual analogue scale from 0% to 100%. Participants had ten seconds to choose a likelihood score with the continuous visual analogue scale. The total experimental procedure lasted less than 35 min. A response box with two buttons was used to move a slider across the visual analogue scale. If no selection was made, the choice “50” (default choice on the scale) was automatically registered for that event.

### Data acquisition and pre-processing

Behavioural analyses were performed in SPSS 25 (International Business Machines Corporation, Armonk, NY, USA). Participants used a visual analogue scale from 0% to 100% to determine the perceived likelihood estimate of a character experiencing a target event. Trials in which participants did not move the slider (approximately 1% of all trials) were excluded from both the behavioural and neuroimaging analyses. The dependent variable was the difference in the z-standardized likelihood estimates between desirable and undesirable events, computed at the level of each participant, separately for each of the four fictional characters.

All MRI images were acquired using a 3 Tesla Siemens Magnetom Prisma Scanner (Siemens, Erlangen, Germany) with a 64-channel head coil at the Insel University Hospital in Bern, Switzerland. Volumes were registered using a T2*-weighted multi-band echo-planar imaging sequence (multi-band EPI) with 48 slices covering the whole brain (slice thickness = 2 mm; 0.5 mm gap; interleaved slice order; TR/TE = 1000 ms/30 ms; flip angle = 80°; field of view = 192 × 192 mm; matrix size = 96 × 96; voxel size = 2 × 2 × 2.5 mm; PAT mode GRAPPA; acceleration factor 2; multiband factor = 3). An anatomical scan (MP-RAGE; 1 mm isotropic voxels; TR = 2300 ms; TE = 2.98 ms; flip angle = 9°; matrix size = 256 × 256) was conducted before the functional run to get highly resolved structural information for the normalization procedure.

Statistical Parametric Mapping software (SPM12; Welcome Department of Cognitive Neurology, London, UK; http://www.fil.ion.ucl.ac.uk/spm) implemented in Matlab R2017b (Mathworks Inc., Sherborn, USA) was used for data analysis. Calculations were performed on UBELIX (https://ubelix.unibe.ch/docs), the high-performance computing cluster at the University of Bern. After slice time correction (middle slice acquisition was used as a reference slice), unwarping and spatial realignment (4th-degree b-Spline interpolation), retrospective noise correction was carried out using the Functional Image Artefact Correction Heuristic Package (FIACH^179^; implemented in R; R Development Core Team, 2008). Moreover, six principal components of physiological noise regressors were calculated with FIACH. Next, functional data were co-registered to each participant’s anatomical image, normalized to the standard space of the Montreal Neurological Institute (MNI) EPI template to permit group analyses, and spatially smoothed with an isotropic three-dimensional Gaussian filter with a full-width at half maximum (FWHM) of 6 mm.

For statistical analyses, event-related signal changes were modelled separately for each participant, using the general linear model (GLM) as implemented in SPM12, in which each trial was modelled with a boxcar function with a variable duration equal to the trial-by-trial deliberation time, i.e. from the start of the trial until the release of the buttons of the response box. For all fMRI analyses, nuisance regressors were included as follows: six movement parameters from the realignment procedure, six physiological noise parameters obtained during noise correction with FIACH. Additionally, a constant covariate representing the session-specific mean over scans was implemented in the first-level model. The models included a high-pass filter of 128 s to remove low-frequency drift of the scanner and first-order auto-regressive corrections for auto-correlation between scans.

A voxel height threshold of p < 0.001 was adopted for whole-brain analyses, with correction for multiple comparisons performed at the cluster level (p < 0.01), as first described by Slotnick *et al*.^180^. The cluster extent threshold procedure relies on the fact that, given spurious activity or noise (voxel-wise type I error), the probability of observing increasingly large (spatially contiguous) clusters of activity systematically decreases^181^. Thus, a cluster extent threshold can be enforced to ensure an acceptable probability of cluster-wise type I error. This approach is more sensitive to small effects than the standard 0.05 familywise error correction, while still being an adequate correction for multiple comparisons^181–183^. Our cluster extent threshold was obtained by simulating whole-brain fMRI activation using custom software written in Matlab^180,184^. The script modelled our entire functional image matrix 94 × 94 × 48 (i.e. acquisition matrix by slice), assumed a type I error voxel activation probability of 0.001, and smoothed the activation map by convolution with a 3-dimensional 6 FWHM Gaussian kernel. After 10,000 Monte Carlo independent iterations, the probability of each cluster size was determined and the cluster extent that yielded P < 0.01 was selected for use in voxel extent thresholding, i.e. 30 contiguous resampled voxels (23 original voxels) or a volume of 240 mm^3^. Each contrast that entered a null conjunction analysis was also thresholded at p < 0.001, with the same cluster extent of 30 voxels as described above.

### Data analysis

To determine how the desirability bias manifests in the brain, we performed a series of brain-behaviour correlation analyses between the SCM group-specific desirability biases, on one hand, and the parameter estimates related to activity within each of the respective set of regions hypothesized (H1, H2, H3 and H4). Specifically, based on our hypotheses, we first determined significant group differences at the brain level. To this aim, we calculated several hypothesis-driven contrasts in the BOLD signal (see Results section for details). We then calculated the mean values of the parameter estimates in a 6 mm cube around the peak voxels determined in the hypothesis-driven contrasts of all trials pertaining to a specific SCM character, irrespective of the valence of the trial (desirable or undesirable). At the behavioural level, we determined desirability bias scores by calculating the difference [estimated likelihood for desirable events – estimated likelihood for undesirable events] for each SCM character in each participant. Finally, we opted for brain-behaviour correlations between these desirability bias scores for each SCM social group, on one hand, and the BOLD signal change in relevant brain regions, on the other hand. For the latter, we calculated the mean values of the parameter estimates in a 6 mm cube around the peak voxels found significant at the group-level in the unmodulated analysis of all trials pertaining to a specific social target group, irrespective of the valence of the trial (desirable or undesirable).

We chose the more robust and powerful “skipped” correlations as suggested by Rousselet and Pernet^174^, and implemented in the Robust Correlation Matlab Toolbox (https://sourceforge.net/projects/robustcorrtool)^173^. Skipped correlations are robust measures of association between variables that provide effect sizes and confidence intervals subsequent to removing outliers. For comparison, we also report traditional Spearman correlation estimates with all potential outliers included, as suggested elsewhere^174^. In those brain-behaviour correlation analyses that comprised the examination of multiple regions, we used the Benjamini-Hochberg step-up procedure to correct for multiple testing (false discovery rate alpha = 0.05, two-tailed^185^). The three sets of brain-behaviour correlations (H1, H2, H3 and H4) were treated as independent from each other for the purposes of this correction.

## Supplementary information

Supplementary Materials.

## Data Availability

Under the Swiss guidelines of data protection (Ordinance HFV Art. 5), the datasets generated and analysed during the current study can be made available from the corresponding author on a case by case basis.

## References

[1] Atance CM, O’Neill DK (2001). Episodic future thinking. Trends in cognitive sciences.

[2] Suddendorf T, Addis DR, Corballis MC (2009). Mental time travel and the shaping of the human mind. Philosophical Transactions of the Royal Society B: Biological Sciences.

[3] Schacter DL, Addis DR, Buckner RL (2007). Remembering the past to imagine the future: the prospective brain. Nature reviews neuroscience.

[4] Stapel DA, Velthuijsen AS (1996). Just as if it happened to me: The impact of vivid and self-relevant information on risk judgments. Journal of Social and Clinical Psychology.

[5] Tyler TR, Cook FL (1984). The mass media and judgments of risk: Distinguishing impact on personal and societal level judgments. Journal of Personality and Social Psychology.

[6] Weinstein ND (1980). Unrealistic optimism about future life events. Journal of Personality and Social Psychology.

[7] Harris P, Middleton W (1994). The illusion of control and optimism about health: On being less at risk but no more in control than others. British Journal of Social Psychology.

[8] Helweg-Larsen M, Shepperd JA (2001). Do moderators of the optimistic bias affect personal or target risk estimates? A review of the literature. Personality and Social Psychology Review.

[9] Tice DM, Butler JL, Muraven MB, Stillwell AM (1995). When modesty prevails: Differential favorability of self-presentation to friends and strangers. Journal of Personality and Social Psychology.

[10] Liviatan I, Trope Y, Liberman N (2008). Interpersonal similarity as a social distance dimension: Implications for perception of others’ actions. Journal of Experimental Social Psychology.

[11] Harris P, Middleton W, Joiner R (2000). The typical student as an in‐group member: eliminating optimistic bias by reducing social distance. European Journal of Social Psychology.

[12] Hoorens V (1995). Self‐favoring biases, self‐presentation, and the self‐other asymmetry in social comparison. Journal of Personality.

[13] Menon G, Kyung EJ, Agrawal N (2009). Biases in social comparisons: Optimism or pessimism?. Organizational Behavior and Human Decision Processes.

[14] Perloff LS, Fetzer BK (1986). Self–other judgments and perceived vulnerability to victimization. Journal of Personality and Social Psychology.

[15] Price PC (2000). Wishful thinking in the prediction of competitive outcomes. Thinking & Reasoning.

[16] Hollander B (2004). People think like me: Religion and wishful thinking in the 2000 US presidential election. Journal of Media and Religion.

[17] Babad E (1997). Wishful thinking among voters: Motivational and cognitive influences. International Journal of Public Opinion Research.

[18] Aue T, Nusbaum HC, Cacioppo JT (2011). Neural correlates of wishful thinking. Social Cognitive and Affective Neuroscience.

[19] Babad, E. Wishful thinking and objectivity among sports fans. *Social Behaviour* (1987).

[20] Krizan Z, Windschitl PD (2007). The influence of outcome desirability on optimism. Psychological Bulletin.

[21] Lench HC (2009). Automatic optimism: The affective basis of judgments about the likelihood of future events. Journal of Experimental Psychology: General.

[22] Chambers JR, Windschitl PD (2004). Biases in social comparative judgments: the role of nonmotivated factors in above-average and comparative-optimism effects. Psychological Bulletin.

[23] Windschitl PD, Rose JP, Stalkfleet MT, Smith AR (2008). Are people excessive or judicious in their egocentrism? A modeling approach to understanding bias and accuracy in people’s optimism. Journal of Personality and Social Psychology.

[24] Alicke MD (1985). Global self-evaluation as determined by the desirability and controllability of trait adjectives. Journal of Personality and Social Psychology.

[25] Gardner WL, Gabriel S, Hochschild L (2002). When you and I are "we", you are not threatening: the role of self-expansion in social comparison. Journal of Personality and Social Psychology.

[26] Windschitl, P. D. & Stuart, J. O. R. *Optimism biases*. (Wiley-Blackwell, 2015).

[27] Castano E, Yzerbyt V, Bourguignon D, Seron E (2002). Who may enter? The impact of in-group identification on in-group/out-group categorization. Journal of Experimental Social Psychology.

[28] Hogg MA, Reid SA (2006). Social identity, self‐categorization, and the communication of group norms. Communication Theory.

[29] Rijswijk W, Haslam SA, Ellemers N (2006). Who do we think we are? The effects of social context and social identification on in‐group stereotyping. British Journal of Social Psychology.

[30] Tajfel, H. & Turner, J. *The social identity theory of intergroup behavior* 7–24 (Nelson-Hall, 1986).

[31] Denny BT, Kober H, Wager TD, Ochsner KN (2012). A meta-analysis of functional neuroimaging studies of self-and other judgments reveals a spatial gradient for mentalizing in medial prefrontal cortex. Journal of cognitive Neuroscience.

[32] Cikara M, Van Bavel JJ, Ingbretsen ZA, Lau T (2017). Decoding “us” and “them”: Neural representations of generalized group concepts. Journal of Experimental Psychology: General.

[33] Tajfel H, Billig MG, Bundy RP, Flament C (1971). Social categorization and intergroup behaviour. European Journal of Social Psychology.

[34] Addis DR, Wong AT, Schacter DL (2007). Remembering the past and imagining the future: common and distinct neural substrates during event construction and elaboration. Neuropsychologia.

[35] Maddock RJ, Garrett AS, Buonocore MH (2001). Remembering familiar people: the posterior cingulate cortex and autobiographical memory retrieval. Neuroscience.

[36] Moran JM, Macrae CN, Heatherton TF, Wyland CL, Kelley WM (2006). Neuroanatomical evidence for distinct cognitive and affective components of self. Journal of cognitive neuroscience.

[37] Morrison S, Decety J, Molenberghs P (2012). The neuroscience of group membership. Neuropsychologia.

[38] Spreng RN, Mar RA, Kim AS (2009). The common neural basis of autobiographical memory, prospection, navigation, theory of mind, and the default mode: a quantitative meta-analysis. Journal of cognitive neuroscience.

[39] Volz KG, Kessler T, von Cramon DY (2009). In-group as part of the self: in-group favoritism is mediated by medial prefrontal cortex activation. Social neuroscience.

[40] Chavez RS, Heatherton TF, Wagner DD (2016). Neural population decoding reveals the intrinsic positivity of the self. Cerebral Cortex.

[41] Macrae CN, Bodenhausen VG (2000). Social Cognition: Thinking Categorically about Others. Annual Review of Psychology.

[42] Quadflieg S, Macrae CN (2011). Stereotypes and stereotyping: What’s the brain got to do with it?. European Review of Social Psychology.

[43] Bonner MF, Price AR (2013). Where Is the Anterior Temporal Lobe and What Does It Do?. The Journal of Neuroscience.

[44] Amodio DM (2014). The neuroscience of prejudice and stereotyping. Nature Reviews Neuroscience.

[45] Heleven E, Van Overwalle F (2018). The neural basis of representing others’ inner states. Current opinion in psychology.

[46] Krall SC (2015). The role of the right temporoparietal junction in attention and social interaction as revealed by ALE meta-analysis. Brain Structure and Function.

[47] Preckel K, Kanske P, Singer T (2018). On the interaction of social affect and cognition: empathy, compassion and theory of mind. Current Opinion in Behavioral Sciences.

[48] Steinbeis N (2016). The role of self–other distinction in understanding others’ mental and emotional states: neurocognitive mechanisms in children and adults. Philosophical Transactions of the Royal Society B: Biological Sciences.

[49] Olson IR, McCoy D, Klobusicky E, Ross LA (2013). Social cognition and the anterior temporal lobes: a review and theoretical framework. Social cognitive and affective neuroscience.

[50] Wong C, Gallate J (2012). The function of the anterior temporal lobe: a review of the empirical evidence. Brain research.

[51] Gilbert SJ, Swencionis JK, Amodio DM (2012). Evaluative vs. trait representation in intergroup social judgments: Distinct roles of anterior temporal lobe and prefrontal cortex. Neuropsychologia.

[52] Gamond L, Ferrari C, La Rocca S, Cattaneo Z (2017). Dorsomedial prefrontal cortex and cerebellar contribution to in‐group attitudes: a transcranial magnetic stimulation study. European Journal of Neuroscience.

[53] Ferrari C, Vecchi T, Todorov A, Cattaneo Z (2016). Interfering with activity in the dorsomedial prefrontal cortex via TMS affects social impressions updating. Cognitive, Affective, & Behavioral Neuroscience.

[54] Gozzi M, Raymont V, Solomon J, Koenigs M, Grafman J (2009). Dissociable effects of prefrontal and anterior temporal cortical lesions on stereotypical gender attitudes. Neuropsychologia.

[55] Abele AE, Wojciszke B (2013). The Big Two in social judgment and behavior. Social Psychology.

[56] Fiske ST, Cuddy AJ, Glick P (2007). Universal dimensions of social cognition: Warmth and competence. Trends in Cognitive Sciences.

[57] Wojciszke B, Abele AE, Baryla W (2009). Two dimensions of interpersonal attitudes: Liking depends on communion, respect depends on agency. European Journal of Social Psychology.

[58] Cuddy AJ, Fiske ST, Glick P (2008). Warmth and competence as universal dimensions of social perception: The stereotype content model and the BIAS map. Advances in Experimental Social Psychology.

[59] Judd CM, James-Hawkins L, Yzerbyt V, Kashima Y (2005). Fundamental dimensions of social judgment: understanding the relations between judgments of competence and warmth. Journal of personality and social psychology.

[60] Cuddy AJ, Fiske ST, Glick P (2007). The BIAS map: behaviors from intergroup affect and stereotypes. Journal of Personality and Social Psychology.

[61] Kervyn, N., Fiske, S. & Yzerbyt, V. Forecasting the primary dimension of social perception. *Social Psychology* (2015). 10.1027/1864-9335/a000219 PMC6294451 30555596

[62] Caprariello PA, Cuddy AJ, Fiske ST (2009). Social structure shapes cultural stereotypes and emotions: A causal test of the stereotype content model. Group Processes & Intergroup Relations.

[63] Wojciszke B, Abele AE (2008). The primacy of communion over agency and its reversals in evaluations. European Journal of Social Psychology.

[64] Cuddy AJ (2009). Stereotype content model across cultures: Towards universal similarities and some differences. British Journal of Social Psychology.

[65] Fiske ST (2015). Intergroup biases: A focus on stereotype content. Current Opinion in Behavioral Sciences.

[66] Cikara M, Fiske TS (2011). Bounded Empathy: Neural Responses to Outgroup Targets’ (Mis)fortunes. Journal of Cognitive Neuroscience.

[67] Cuddy AJ, Norton MI, Fiske ST (2005). This old stereotype: The pervasiveness and persistence of the elderly stereotype. Journal of Social Issues.

[68] Fiske ST (2017). Prejudices in cultural contexts: shared stereotypes (gender, age) versus variable stereotypes (race, ethnicity, religion). Perspectives on Psychological Science.

[69] Harris, L. T., Cikara, M. & Fiske, S. T. *Envy as predicted by the stereotype content model: Volatile ambivalence*. (Oxford University Press., 2008).

[70] Fiske ST (2012). Managing ambivalent prejudices: smart-but-cold and warm-but-dumb stereotypes. The Annals of the American Academy of Political and Social Science.

[71] Harris LT, Fiske ST (2006). Dehumanizing the lowest of the low: Neuroimaging responses to extreme out-groups. Psychological science.

[72] Harris LT, Fiske ST (2007). Social groups that elicit disgust are differentially processed in mPFC. Social cognitive and affective neuroscience.

[73] Cikara M, Van Bavel JJ (2014). The Neuroscience of Intergroup Relations:An Integrative Review. Perspectives on Psychological Science.

[74] Dricu M (2018). Warmth and competence predict overoptimistic beliefs for out-group but not in-group members. PloS one.

[75] Northoff G (2006). Self-referential processing in our brain—a meta-analysis of imaging studies on the self. Neuroimage.

[76] Fiske ST (2013). Divided by status: Upward envy and downward scorn. Proceedings of the American Philosophical Society.

[77] Cikara M, Fiske ST (2012). Stereotypes and schadenfreude: Affective and physiological markers of pleasure at outgroup misfortunes. *Social Psychological and Personality*. Science.

[78] Fehse K, Silveira S, Elvers K, Blautzik J (2015). Compassion, guilt and innocence: an fMRI study of responses to victims who are responsible for their fate. Social neuroscience.

[79] Gerdes KE (2011). Empathy, sympathy, and pity: 21st-century definitions and implications for practice and research. Journal of Social Service Research.

[80] Goetz JL, Keltner D, Simon-Thomas E (2010). Compassion: an evolutionary analysis and empirical review. Psychological bulletin.

[81] Mascaro JS, Rilling JK, Tenzin Negi L, Raison CL (2012). Compassion meditation enhances empathic accuracy and related neural activity. Social cognitive and affective neuroscience.

[82] Singer T, Klimecki OM (2014). Empathy and compassion. Current Biology.

[83] Cikara M, Farnsworth RA, Harris LT, Fiske ST (2010). On the wrong side of the trolley track: Neural correlates of relative social valuation. Social cognitive and affective neuroscience.

[84] Lee TL, Fiske ST (2006). Not an outgroup, not yet an ingroup: Immigrants in the stereotype content model. International Journal of Intercultural Relations.

[85] Lamm C, Decety J, Singer T (2011). Meta-analytic evidence for common and distinct neural networks associated with directly experienced pain and empathy for pain. Neuroimage.

[86] Lutz A, Brefczynski-Lewis J, Johnstone T, Davidson RJ (2008). Regulation of the neural circuitry of emotion by compassion meditation: effects of meditative expertise. PloS one.

[87] Singer T, Critchley HD, Preuschoff K (2009). A common role of insula in feelings, empathy and uncertainty. Trends in cognitive sciences.

[88] Marcoux, L.-A., Michon, P.-E., Voisin, J., Lemelin, S. & Jackson, P. The modulation of somatosensory resonance by psychopathic traits and empathy. *Frontiers in Human Neuroscience* **7**, 10.3389/fnhum.2013.00274 (2013). 10.3389/fnhum.2013.00274 PMC3685719 23801950

[89] Singer T, Lamm C (2009). The social neuroscience of empathy. Annals of the New York Academy of Sciences.

[90] Kross E, Berman MG, Mischel W, Smith EE, Wager TD (2011). Social rejection shares somatosensory representations with physical pain. Proceedings of the National Academy of Sciences.

[91] Silani G, Lamm C, Ruff CC, Singer T (2013). Right supramarginal gyrus is crucial to overcome emotional egocentricity bias in social judgments. Journal of neuroscience.

[92] Balconi M, Bortolotti A (2012). Detection of the facial expression of emotion and self-report measures in empathic situations are influenced by sensorimotor circuit inhibition by low-frequency rTMS. Brain Stimulation.

[93] Jospe K, Flöel A, Lavidor M (2018). The interaction between embodiment and empathy in facial expression recognition. Social cognitive and affective neuroscience.

[94] Harris LT, Fiske ST (2011). Dehumanized perception: A psychological means to facilitate atrocities, torture, and genocide?. Zeitschrift für Psychologie/Journal of Psychology.

[95] Moll J (2005). The moral affiliations of disgust: A functional MRI study. Cognitive and behavioral neurology.

[96] Schienle A, Schäfer A, Stark R, Walter B, Vaitl D (2005). Gender differences in the processing of disgust-and fear-inducing pictures: an fMRI study. Neuroreport.

[97] Molenberghs P (2013). The neuroscience of in-group bias. Neuroscience & Biobehavioral Reviews.

[98] Brewer MB (1999). The psychology of prejudice: Ingroup love and outgroup hate?. Journal of social issues.

[99] Alicke MD, Klotz ML, Breitenbecher DL, Yurak TJ, Vredenburg DS (1995). Personal contact, individuation, and the better-than-average effect. Journal of Personality and Social Psychology.

[100] Poldrack RA (2006). Can cognitive processes be inferred from neuroimaging data?. Trends in cognitive sciences.

[101] Poldrack RA (2011). Inferring mental states from neuroimaging data: from reverse inference to large-scale decoding. Neuron.

[102] van Veelen R, Otten S, Hansen N (2013). Social identification when an in‐group identity is unclear: The role of self‐anchoring and self‐stereotyping. British Journal of Social Psychology.

[103] Kleiman EM (2017). Optimism and well-being: A prospective multi-method and multi-dimensional examination of optimism as a resilience factor following the occurrence of stressful life events. Cognition and Emotion.

[104] Reynolds KJ, Turner JC, Haslam SA (2000). When are we better than them and they worse than us? A closer look at social discrimination in positive and negative domains. Journal of Personality and Social Psychology.

[105] Klimecki OM, Leiberg S, Lamm C, Singer T (2012). Functional neural plasticity and associated changes in positive affect after compassion training. Cerebral cortex.

[106] Xiong R-C (2019). Brain pathways of pain empathy activated by pained facial expressions: a meta-analysis of fMRI using the activation likelihood estimation method. Neural regeneration research.

[107] Timmers I (2018). Is empathy for pain unique in its neural correlates? A meta-analysis of neuroimaging studies of empathy. Frontiers in behavioral neuroscience.

[108] Fox KC (2016). Functional neuroanatomy of meditation: A review and meta-analysis of 78 functional neuroimaging investigations. Neuroscience & Biobehavioral Reviews.

[109] Hofmann SG, Grossman P, Hinton DE (2011). Loving-kindness and compassion meditation: Potential for psychological interventions. Clinical psychology review.

[110] Avenanti A, Bueti D, Galati G, Aglioti SM (2005). Transcranial magnetic stimulation highlights the sensorimotor side of empathy for pain. Nature neuroscience.

[111] Avenanti A, Paluello IM, Bufalari I, Aglioti SM (2006). Stimulus-driven modulation of motor-evoked potentials during observation of others’ pain. Neuroimage.

[112] Lockwood PL, Iannetti GD, Haggard P (2013). Transcranial magnetic stimulation over human secondary somatosensory cortex disrupts perception of pain intensity. cortex.

[113] Vandenbroucke S, Bardi L, Lamm C, Goubert L (2016). The role of the right temporoparietal junction in the elicitation of vicarious experiences and detection accuracy while observing pain and touch. Experimental brain research.

[114] Lehner R, Meesen R, Wenderoth N (2017). Observing back pain provoking lifting actions modulates corticomotor excitability of the observer’s primary motor cortex. Neuropsychologia.

[115] Coll M-P, Tremblay M-PB, Jackson PL (2017). The effect of tDCS over the right temporo-parietal junction on pain empathy. Neuropsychologia.

[116] Mai X (2016). Using tDCS to explore the role of the right temporo-parietal junction in theory of mind and cognitive empathy. Frontiers in psychology.

[117] Robinson C (2019). Modulating affective experience and emotional intelligence with loving kindness meditation and transcranial direct current stimulation: a pilot study. Social neuroscience.

[118] Santiesteban I, Banissy MJ, Catmur C, Bird G (2012). Enhancing social ability by stimulating right temporoparietal junction. Current Biology.

[119] Sellaro R (2015). Increasing the role of belief information in moral judgments by stimulating the right temporoparietal junction. Neuropsychologia.

[120] Riek BM, Mania EW, Gaertner SL (2006). Intergroup threat and outgroup attitudes: A meta-analytic review. Personality and social psychology review.

[121] IJzerman H, Van Prooijen J-W (2008). Just world and the emotional defense of self. Social Psychology.

[122] Alves, H. & Correia, I. The buffering-boosting hypothesis of the expression of general and personal belief in a just world for successes and failures. *Social Psychology* (2013).

[123] Bardi L, Six P, Brass M (2017). Repetitive TMS of the temporo-parietal junction disrupts participant’s expectations in a spontaneous Theory of Mind task. Social cognitive and affective neuroscience.

[124] Baumgartner T, Schiller B, Rieskamp J, Gianotti LR, Knoch D (2013). Diminishing parochialism in intergroup conflict by disrupting the right temporo-parietal junction. Social cognitive and affective neuroscience.

[125] Costa A, Torriero S, Oliveri M, Caltagirone C (2008). Prefrontal and temporo-parietal involvement in taking others’ perspective: TMS evidence. Behavioural neurology.

[126] Paracampo R, Pirruccio M, Costa M, Borgomaneri S, Avenanti A (2018). Visual, sensorimotor and cognitive routes to understanding others’ enjoyment: An individual differences rTMS approach to empathic accuracy. Neuropsychologia.

[127] Soutschek A, Ruff CC, Strombach T, Kalenscher T, Tobler PN (2016). Brain stimulation reveals crucial role of overcoming self-centeredness in self-control. Science advances.

[128] Young L, Camprodon JA, Hauser M, Pascual-Leone A, Saxe R (2010). Disruption of the right temporoparietal junction with transcranial magnetic stimulation reduces the role of beliefs in moral judgments. Proceedings of the National Academy of Sciences.

[129] Young L, Cushman F, Hauser M, Saxe R (2007). The neural basis of the interaction between theory of mind and moral judgment. Proceedings of the National Academy of Sciences.

[130] Donaldson PH, Kirkovski M, Rinehart NJ, Enticott PG (2018). Autism‐relevant traits interact with temporoparietal junction stimulation effects on social cognition: a high‐definition transcranial direct current stimulation and electroencephalography study. European Journal of Neuroscience.

[131] Koster-Hale J (2017). Mentalizing regions represent distributed, continuous, and abstract dimensions of others’ beliefs. Neuroimage.

[132] Tusche A, Böckler A, Kanske P, Trautwein F-M, Singer T (2016). Decoding the charitable brain: empathy, perspective taking, and attention shifts differentially predict altruistic giving. Journal of Neuroscience.

[133] Lamm C, Bukowski H, Silani G (2016). From shared to distinct self–other representations in empathy: evidence from neurotypical function and socio-cognitive disorders. Philosophical Transactions of the Royal Society B: Biological Sciences.

[134] Vossel S, Geng JJ, Fink GR (2014). Dorsal and ventral attention systems: distinct neural circuits but collaborative roles. The Neuroscientist.

[135] Parkinson C, Wheatley T (2013). Old cortex, new contexts: Re-purposing spatial perception for social cognition. Frontiers in human neuroscience.

[136] Binney RJ, Embleton KV, Jefferies E, Parker GJ, Lambon Ralph MA (2010). The ventral and inferolateral aspects of the anterior temporal lobe are crucial in semantic memory: evidence from a novel direct comparison of distortion-corrected fMRI, rTMS, and semantic dementia. Cerebral Cortex.

[137] Busigny T (2014). Face-specific impairment in holistic perception following focal lesion of the right anterior temporal lobe. Neuropsychologia.

[138] Evans JJ, Heggs A, Antoun N, Hodges JR (1995). Progressive prosopagnosia associated with selective right temporal lobe atrophy: A new syndrome?. Brain.

[139] Gainotti G, Barbier A, Marra C (2003). Slowly progressive defect in recognition of familiar people in a patient with right anterior temporal atrophy. Brain.

[140] Gentileschi V, Sperber S, Spinnler H (2001). Crossmodal agnosia for familiar people as a consequence of right infero-polar temporal atrophy. Cognitive Neuropsychology.

[141] Lambon Ralph MA, Cipolotti L, Manes F, Patterson K (2010). Taking both sides: do unilateral anterior temporal lobe lesions disrupt semantic memory?. Brain.

[142] Schwartz MF (2009). Anterior temporal involvement in semantic word retrieval: voxel-based lesion-symptom mapping evidence from aphasia. Brain.

[143] Thompson SA, Patterson K, Hodges JR (2003). Left/right asymmetry of atrophy in semantic dementia: behavioral–cognitive implications. Neurology.

[144] Campanella F, Fabbro F, Urgesi C (2013). Cognitive and anatomical underpinnings of the conceptual knowledge for common objects and familiar people: a repetitive transcranial magnetic stimulation study. PloS one.

[145] Chiou R, Sowman PF, Etchell AC, Rich AN (2014). A conceptual lemon: theta burst stimulation to the left anterior temporal lobe untangles object representation and its canonical color. Journal of cognitive neuroscience.

[146] Gallate J, Wong C, Ellwood S, Chi R, Snyder A (2011). Noninvasive brain stimulation reduces prejudice scores on an implicit association test. Neuropsychology.

[147] Hoffman P, Crutch S (2016). Knowing what and where: TMS evidence for the dual neural basis of geographical knowledge. cortex.

[148] Pobric G, Jefferies E, Ralph MAL (2007). Anterior temporal lobes mediate semantic representation: mimicking semantic dementia by using rTMS in normal participants. Proceedings of the National Academy of Sciences.

[149] Pobric G, Jefferies E, Ralph MAL (2010). Category-specific versus category-general semantic impairment induced by transcranial magnetic stimulation. Current Biology.

[150] Pobric G, Jefferies E, Ralph MAL (2010). Amodal semantic representations depend on both anterior temporal lobes: evidence from repetitive transcranial magnetic stimulation. Neuropsychologia.

[151] Pobric G, Ralph MAL, Jefferies E (2009). The role of the anterior temporal lobes in the comprehension of concrete and abstract words: rTMS evidence. Cortex.

[152] Wong C, Gallate J (2011). Low-frequency repetitive transcranial magnetic stimulation of the anterior temporal lobes does not dissociate social versus nonsocial semantic knowledge. The Quarterly Journal of Experimental Psychology.

[153] Wong CL, Harris JA, Gallate JE (2012). Evidence for a social function of the anterior temporal lobes: Low-frequency rTMS reduces implicit gender stereotypes. Social neuroscience.

[154] Woollams AM (2012). Apples are not the only fruit: the effects of concept typicality on semantic representation in the anterior temporal lobe. Frontiers in Human Neuroscience.

[155] Platonov A (2019). Rapid and specific processing of person-related information in human anterior temporal lobe. Communications biology.

[156] Irish M, Hodges JR, Piguet O (2014). Right anterior temporal lobe dysfunction underlies theory of mind impairments in semantic dementia. Brain.

[157] Zahn R (2009). Social conceptual impairments in frontotemporal lobar degeneration with right anterior temporal hypometabolism. Brain.

[158] Caruana F, Jezzini A, Sbriscia-Fioretti B, Rizzolatti G, Gallese V (2011). Emotional and social behaviors elicited by electrical stimulation of the insula in the macaque monkey. Current Biology.

[159] Papagno C (2016). Specific disgust processing in the left insula: new evidence from direct electrical stimulation. Neuropsychologia.

[160] Ponz A (2013). Emotion processing in words: a test of the neural re-use hypothesis using surface and intracranial EEG. Social Cognitive and Affective Neuroscience.

[161] Ottaviani C, Mancini F, Provenzano S, Collazzoni A, D’Olimpio F (2018). Deontological morality can be experimentally enhanced by increasing disgust: A transcranial direct current stimulation study. Neuropsychologia.

[162] Ziegler JC (2018). Do words stink? Neural reuse as a principle for understanding emotions in reading. Journal of cognitive neuroscience.

[163] Heekeren HR, Marrett S, Ruff DA, Bandettini P, Ungerleider LG (2006). Involvement of human left dorsolateral prefrontal cortex in perceptual decision making is independent of response modality. Proceedings of the National Academy of Sciences.

[164] Pessoa L, Padmala S (2005). Quantitative prediction of perceptual decisions during near-threshold fear detection. Proceedings of the National Academy of Sciences.

[165] Keuken, M. C. *et al*. Brain networks of perceptual decision-making: an fMRI ALE meta-analysis. *Frontiers in Human Neuroscience* **8**, 10.3389/fnhum.2014.00445 (2014). 10.3389/fnhum.2014.00445 PMC4063192 24994979

[166] Hauser, C. K. & Salinas, E. Perceptual Decision-Making. *Encyclopedia of Computational Neuroscience*, 2243–2261 (2015).

[167] Sedda A, Scarpina F (2012). Dorsal and ventral streams across sensory modalities. Neuroscience bulletin.

[168] Summerfield C (2006). Predictive codes for forthcoming perception in the frontal cortex. Science.

[169] Takahashi E, Ohki K, Kim D-S (2013). Dissociation and convergence of the dorsal and ventral visual working memory streams in the human prefrontal cortex. NeuroImage.

[170] Wendelken C, Ditterich J, Bunge SA, Carter CS (2009). Stimulus and response conflict processing during perceptual decision making. Cognitive, Affective, & Behavioral Neuroscience.

[171] Yarkoni, T. & Braver, T. S. In *Handbook of individual differences in cognition* 87–107 (Springer, 2010).

[172] Yarkoni T (2009). Big correlations in little studies: Inflated fMRI correlations reflect low statistical power—Commentary on Vul et al.(2009). Perspectives on Psychological Science.

[173] Pernet, C., Wilcox, R. & Rousselet, G. Robust Correlation Analyses: False Positive and Power Validation Using a New Open Source Matlab Toolbox. *Frontiers in Psychology* **3**, 10.3389/fpsyg.2012.00606 (2013). 10.3389/fpsyg.2012.00606 PMC3541537 23335907

[174] Rousselet, G. & Pernet, C. Improving standards in brain-behavior correlation analyses. *Frontiers in Human Neuroscience* **6**, 10.3389/fnhum.2012.00119 (2012). 10.3389/fnhum.2012.00119 PMC3342588 22563313

[175] Wood, G., Willmes, K., Nuerk, H.-C. & Fischer, M. H. On the cognitive link between space and number: a meta-analysis of the SNARC effect. *Psychology Science* (2008).

[176] Mišić B, Sporns O (2016). From regions to connections and networks: new bridges between brain and behavior. Current opinion in neurobiology.

[177] O’Reilly JX, Woolrich MW, Behrens TE, Smith SM, Johansen-Berg H (2012). Tools of the trade: psychophysiological interactions and functional connectivity. Social cognitive and affective neuroscience.

[178] Kotter-Grühn D, Hess TM (2012). The impact of age stereotypes on self-perceptions of aging across the adult lifespan. Journals of Gerontology Series B: Psychological Sciences and Social Sciences.

[179] Tierney TM (2016). FIACH: a biophysical model for automatic retrospective noise control in fMRI. Neuroimage.

[180] Slotnick SD, Moo LR, Segal JB, Hart J. (2003). Distinct prefrontal cortex activity associated with item memory and source memory for visual shapes. Cognitive Brain Research.

[181] Slotnick SD, Schacter DL (2004). A sensory signature that distinguishes true from false memories. Nature neuroscience.

[182] Slotnick SD (2017). Cluster success: fMRI inferences for spatial extent have acceptable false-positive rates. Cognitive neuroscience.

[183] Iordan A, Dolcos S, Dolcos F (2018). Brain activity and network interactions in the impact of internal emotional distraction. Cerebral Cortex.

[184] Slotnick, S. D. *Scripts and Stimuli. Retrieved from* https://www2.bc.edu/sd-slotnick/scripts.htm, (n.d)).

[185] Benjamini Y, Hochberg Y (1995). Controlling the false discovery rate: a practical and powerful approach to multiple testing. Journal of the Royal statistical society: series B (Methodological).

