# Supplementary material for: Group membership dictates the neural correlates of social optimism biases
Source: Sci Rep. 2020 Jan 24;10:1139. doi: 10.1038/s41598-020-58121-4 (PMC6981267; doi:10.1038/s41598-020-58121-4)
Supplement: Supplementary file 1 — Supplementary Materials. [file 41598_2020_58121_MOESM1_ESM.docx]

**Scientific Reports**

**Supplementary information**

Group membership dictates the neural correlates of social optimism biases

Mihai Dricu, Laurent Schüpbach, Mirko Bristle, Roland Wiest, Dominik A. Moser, Tatjana Aue

| **Region** | **Cluster size** | **z-score** | **MNI coordinates** |
| --- | --- | --- | --- |
| Secondary somatosensory cortex | 1626 | 4.96 | 48; -34; 26 |
|  | ^ | 4.9 | 58; -28; 18 |
| Primary somatosensory cortex | ^ | 4.87 | 26; -38; 62 |
| Superior frontal gyrus | 170 | 4.67 | 26; 16; 56 |
| Primary somatosensory cortex | 202 | 4.48 | -18; -26; 68 |
|  | ^ | 4.15 | -18; -44; 62 |
|  | ^ | 3.64 | -24; -34; 68 |
| Secondary somatosensory cortex | 255 | 4.44 | -58; -32; 24 |
|  | ^ | 3.59 | -50; -34; 14 |
| Inferior temporal gyrus | 79 | 4.43 | -56; -58; -8 |
| Precuneus | 42 | 4.32 | -14; -34; 40 |
| Mid cingulate cortex | 85 | 4.12 | 2; -2; 40 |
|  | ^ | 3.68 | 8; -10; 40 |
|  | ^ | 3.5 | 2; 6; 38 |
| Posterior insula | 159 | 4.09 | 52; -4; 4 |
|  | ^ | 3.63 | 42; -10; -8 |
| Middle frontal gyrus | 103 | 4.08 | 38; 46; 20 |
| Cuneus | 27 | 3.93 | -8; -84; 30 |
| Precuneus | 45 | 3.93 | 10; -42; 54 |
|  | ^ | 3.53 | 8; -34; 54 |
| Posterior insula | 77 | 3.86 | -32; -20; 6 |
|  | ^ | 3.64 | -40; -14; -8 |
| Superior frontal gyrus | 130 | 3.73 | -24; 16; 58 |
|  | ^ | 3.64 | -26; 26; 46 |
|  | ^ | 3.48 | -20; 10; 66 |
| Posterior insula | 25 | 3.57 | 32; 6; 6 |

**Table S1**. Regions that are significantly more active when evaluating the warm out-group compared to the cold out-groups, following the pairwise comparison 2*(Mild out-group vs. in-group) - [(Moderate out-group – In-group) + (Extreme out-group – in-group] within the one-way ANOVA with factor minuend character (mild out-group, moderate out-group, extreme out-group) computed on the three second-level contrasts). p < .001 and a cluster-level p < .01. ^ part of the same functional cluster

| **Region** | **Cluster size** | **z-score** | **MNI coordinates** |
| --- | --- | --- | --- |
| Temporo-parietal junction | 341 | 4.84 | 64; -54; 14 |
|  | ^ | 4.69 | 48; -56; 14 |
|  | ^ | 4.19 | 56; -64; 14 |
| Fusiform gyrus | 268 | 4.83 | 40; -42; -20 |
|  | ^ | 4.47 | 42; -56; -18 |
| Anterior temporal lobe | 193 | 4.61 | 48; 12; -36 |
|  | ^ | 3.86 | 40; 22; -26 |
| Occipital lobe | 54 | 4.58 | -28; -78; -8 |
| Mid cingulate cortex | 41 | 4.58 | -2; -26; 26 |
| Superior temporal sulcus | 85 | 4.4 | 48; -20; -10 |
|  | ^ | 3.55 | 44; -34; 0 |
| Precuneus | 60 | 4.22 | -10; -72; 34 |
| Precentral gyrus | 151 | 4.19 | -36; -18; 54 |
|  | ^ | 3.77 | -44; -26; 48 |
| Fusiform gyrus | 47 | 4.09 | -24; -48; -12 |
| Superior temporal sulcus | 24 | 4.04 | -50; -10; -14 |
| Precuneus | 100 | 3.88 | 16; -64; 32 |
|  | ^ | 3.46 | 6; -58; 34 |
|  | ^ | 3.4 | 18; -72; 38 |
| Dorsomedial frontal cortex | 66 | 3.87 | 6; 50; 34 |
| Superior frontal gyrus | 26 | 3.73 | -22; -4; 52 |
| Occipital lobe | 34 | 3.73 | -50; -76; 4 |
| Superior temporal sulcus | 46 | 3.51 | 52; -6; -18 |
|  | ^ | 3.44 | 60; -8; -22 |

**Table S2**. Regions that are significantly more active when evaluating the cold out-groups vs. the warm out-group following the pairwise comparison [(moderate out-group – in-group) + (extreme out-group – in-group)]- 2 *(mild out-group – in-group) within the one-way ANOVA with factor minuend character (mild out-group, moderate out-group, extreme out-group) computed on the three second-level contrasts). p < .001 and a cluster-level p < .01. ^ part of the same functional cluster

| **Region** | **Cluster size** | **z-score** | **MNI coordinates** |
| --- | --- | --- | --- |
| **(In-group–mild out-group)∩(In-group–moderate out-group)∩(In-group–extreme out-group)** | | | |
| - | - | - | - |
| **2 *(Mild out-group–in-group)–[(Moderate out-group–in-group)+(Extreme out-group–in-group)]** | | | |
| Inferior temporal gyrus | 62 | 4.15 | -54, -50, -14 |
| Precentral gyrus | 40 | 3.94 | -40, 4, 26 |
| Middle frontal gyrus | 45 | 3.88 | -26, 24, 46 |
| Middle occipital gyrus | 75 | 3.59 | -38, -78, 36 |
|  | ^ | 3.58 | -32, -84, 38 |
|  | ^ | 3.21 | -28, -78, 42 |
| Middle frontal gyrus | 39 | 3.56 | 28, 16, 58 |
|  | ^ | 3.30 | 30, 6, 52 |
| Precentral gyrus | 30 | 3.50 | -52, 6, 38 |
| **2 *( Extreme out-group –in-group)–[(Moderate out-group–in-group)+(Mild out-group–in-group)]** | | | |
| Dorsomedial prefrontal cortex | 374 | 4.85 | 8, 46, 36 |
|  | ^ | 3.92 | -6, 50, 26 |
|  | ^ | 3.73 | -6, 54, 14 |
| Anterior insula | 32 | 4.07 | -28, 18, -16 |
| Anterior insula | 51 | 4.00 | 38, 28, 2 |
| Anterior insula | 27 | 3.87 | -38, 22, 2 |
| IFC part orbitalis | 44 | 3.82 | 36, 20, -24 |
| Middle temporal gyrus | 27 | 3.40 | 56, -30, -2 |
| **(Moderate out-group–in-group) ∩ (Extreme out-group – in-group)** | | | |
| IFC pars triangularis | 122 | 4.64 | 60, 22, 8 |
|  | ^ | 4.25 | 58, 30, 10 |
| Anterior temporal lobe | 160 | 4.57 | 48, 10, -36 |
|  | ^ | 3.97 | 46, 18, -38 |
| IFC pars orbitalis | 179 | 4.13 | 32, 20, -18 |
|  | ^ | 4.00 | 44, 24, -14 |
|  | ^ | 3.69 | 36, 28, -14 |
| Dorsomedial frontal cortex | 26 | 3.66 | 8, 42, 46 |
|  | ^ | 3.64 | 10, 34, 48 |
| Dorsomedial prefrontal cortex | 92 | 3.61 | 8, 56, 22 |
|  | ^ | 3.54 | -4, 44, 40 |
|  | ^ | 3.18 | 2, 50, 36 |

**Table S3**. Results for desirable events only. Top row depicts the three-way conjunction analysis between in-group and each of the three out-groups. Following rows depict pairwise comparisons for the one-way ANOVA with factor minuend character (mild out-group, moderate out-group, extreme out-group) computed on the three second-level contrasts. p < .001 and a cluster-level p < .01. ^ part of the same functional cluster

| **Region** | **Cluster size** | **z-score** | **MNI coordinates** |
| --- | --- | --- | --- |
| **(In-group – mild out-group) ∩ (In-group – Moderate out-group) ∩ (In-group – extreme out-group)** | | | |
| Precuneus/posterior cingulate cortex | 120 | 4.83 | -2, -54, 20 |
| Ventromedial prefrontal cortex | 134 | 4.06 | -8, 44, -6 |
|  |  | 3.99 | -6, 52, -4 |
|  |  |  |  |
| **2 * (Mild out-group – In-group) – [(Moderate out-group – In-group) + (Extreme out-group – In-group)]** | | | |
| Lingual gyrus | 59 | 3.88 | -12, -66, -8 |
|  |  | 3.75 | -4, -70, 2 |
| Superior frontal gyrus | 53 | 3.75 | -24, 22, 52 |
|  |  |  |  |
| **2 * (Extreme out-group – In-group) – [(Moderate out-group – In-group) + (Mild out-group – In-group)]** | | | |
| Precuneus | 559 | 5.49 | -6, -70, 38 |
|  |  | 4.49 | 12, -72, 42 |
|  |  | 4.47 | 18, -60, 30 |
| Anterior insula | 141 | 5.30 | -30, 14, -16 |
|  |  | 3.73 | -32, 22, -12 |
| Parietal cortex | 204 | 4.18 | -30, -58, 38 |
|  |  | 3.80 | -38, -54, 48 |
| mid cingulate cortex | 62 | 3.98 | -2, -28, 26 |
|  |  | 3.62 | 4, -22, 26 |
| Orbitofrontal cortex | 26 | 3.74 | 18, 28, -22 |
| IFC pars triangularis | 101 | 3.60 | 48, 34, 10 |
|  |  | 3.56 | 46, 24, 22 |
|  |  | 3.47 | 42, 30, 18 |
| Middle frontal gyrus | 60 | 3.59 | -50, 32, 26 |
|  |  | 3.56 | -40, 26, 22 |
|  |  |  |  |
| **(Moderate out-group – In-group) ∩ (Extreme out-group – In-group)** | | | |
| - | - | - | - |

**Table S4**. Results for undesirable events only. Top row depicts the three-way conjunction analysis between in-group and each of the three out-groups. Following rows depict pairwise comparisons for the one-way ANOVA with factor minuend character (mild out-group, moderate out-group, extreme out-group) computed on the three second-level contrasts. p < .001 and a cluster-level p < .01. ^ part of the same functional cluster

| **Main effect of character** | | | |
| --- | --- | --- | --- |
| **Region** | **Cluster size** | **z-score** | **MNI coordinates** |
| Precuneus | 193 | 6.03 | -4, -56, 18 |
| Anterior temporal pole | 211 | 5.96 | 50, 10, -38 |
| Anterior insula | 238 | 5.35 | -32, 18, -16 |
| Dorsomedial frontal cortex | 350 | 5.17 | 6, 52, 30 |
| Ventromedial prefrontal cortex | 150 | 4.94 | -8, 50, -8 |
| Superior frontal gyrus | 168 | 4.87 | 26, 18, 56 |
| IFC – pars triangularis | 160 | 4.86 | 58, 20, 4 |
| IFC – pars orbitalis | 206 | 4.58 | 34, 22, -18 |
| Postcentral gyrus | 177 | 4.55 | 26, -38, 64 |
| Fusiform gyrus | 141 | 4.54 | 42, -42, -16 |
| Inferior temporal gyrus | 56 | 4.40 | -56, -58, -8 |
| Superior temporal gyrus | 54 | 4.27 | 66, -24, 6 |
| Precuneus | 68 | 4.24 | -10, -72, 34 |
| Postcentral gyrus | 83 | 4.16 | 30, -28, 62 |
| Inferior temporal gyrus | 41 | 4.10 | 58, -46, -16 |
| Temporoparietal gyrus | 294 | 4.09 | 46, -56, 20 |
| Supramarginal gyrus | 88 | 3.99 | 54, -28, 18 |
| Pre-supplementary motor cortex | 50 | 3.79 | 4, 26, 52 |
| Supramarginal gyrus | 74 | 3.75 | 50, -42, 50 |
| Superior temporal gyrus | 67 | 3.67 | 52, -2, 0 |
| Superior frontal gyrus | 45 | 3.62 | -24, 20, 56 |
| **Main effect of valence** | | | |
| **Region** | **Cluster size** | **z-score** | **MNI coordinates** |
| Precentral gyrus | 1942 | 7.78 | -34, -22, 52 |
| Fusiform gyrus | 4209 | 7.50 | -22, -70, -8 |
| Anterior temporal pole | 518 | 7.02 | 62, -6, -20 |
| Temporoparietal junction | 1039 | 6.95 | 52, -64, 16 |
| Middle temporal gyrus | 345 | 6.67 | -64, -10, -16 |
| Pre-supplementary motor cortex | 686 | 6.63 | -4, 6, 52 |
| Middle temporal gyrus | 174 | 6.53 | -56, -60, -2 |
| Anterior temporal pole | 193 | 6.19 | 42, 16, -36 |
| Orbitofrontal cortex | 279 | 6.08 | -28, 38, -14 |
| Posterior insula | 454 | 6.01 | -38, -4, -12 |
| Parietal cortex | 857 | 5.81 | -18, -64, 54 |
| Insula | 283 | 5.81 | 40, 2, -12 |
| IFC – pars triangularis | 448 | 5.63 | 50, 38, 4 |
| Temporoparietal junction | 492 | 5.41 | -50, -70, 26 |
| Amygdala | 107 | 5.39 | -20, 8, -26 |
| Occipital cortex | 177 | 5.38 | 30, -76, 22 |
| Supramarginal gyrus | 847 | 5.37 | 62, -38, 36 |
| Dorsomedial prefrontal cortex | 317 | 5.29 | 6, 66, 16 |
| Supramarginal gyrus | 858 | 5.29 | -64, -36, 36 |
| Precentral gyrus | 226 | 5.05 | 60, 12, 0 |
| Ventromedial prefrontal gyrus | 546 | 4.99 | 4, 56, -10 |
| Intraparietal sulcus | 183 | 4.94 | 26, -54, 50 |
| Middle temporal gyrus | 140 | 4.79 | 54, -56, -2 |
| IFC – pars opercularis | 243 | 4.53 | 42, 12, 26 |
| Dorsal anterior cingulate cortex | 64 | 4.51 | 4, 20, 22 |
| Postcentral gyrus | 162 | 4.49 | 26, -36, 60 |
| Postcentral gyrus | 45 | 4.46 | 46, -26, 56 |
| Superior frontal gyrus | 152 | 4.45 | 22, 56, 26 |
| Anterior cingulate cortex | 108 | 4.35 | -4, 34, 10 |
| Occipital cortex | 116 | 4.30 | -26, -82, 18 |
| Superior frontal gyrus | 130 | 4.24 | 20, 24, 50 |
| Middle frontal gyrus | 57 | 4.16 | -48, 44, 14 |
| Superior frontal gyrus | 71 | 4.15 | -18, 30, 50 |
| Postcentral gyrus | 42 | 4.07 | -56, -6, 8 |
| Dorsomedial frontal cortex | 112 | 4.04 | -2, 32, 50 |
| Fusiform gyrus | 40 | 4.04 | 42, -18, -36 |
| Orbitofrontal gyrus | 72 | 4.03 | -16, 52, -18 |
| Fusiform gyrus | 88 | 4.02 | -40, -60, -14 |
| Ventromedial frontal cortex | 40 | 4.00 | -10, 64, 4 |
| Superior frontal gyrus | 54 | 3.85 | -22, 50, 26 |
| IFC – pars opercularis | 75 | 3.82 | -36, 16, 24 |
| IFC – pars orbitalis | 77 | 3.81 | -50, 44, -8 |
| **Interaction effect character x valence** | | | |
| **Region** | **Cluster size** | **z-score** | **MNI coordinates** |
| Anterior temporal lobe | 67 | 4.87 | 46, 12, -32 |
| Pre-supplementary motor cortex | 65 | 4.87 | -6, 0, 64 |
| Postcentral gyrus | 79 | 4.80 | -52, -22, 56 |
| Dorsomedial prefrontal cortex | 206 | 4.67 | 8, 50, 30 |
| Dorsal anterior cingulate cortex | 355 | 4.61 | 2, 30, 26 |
| Superior temporal sulcus | 169 | 4.54 | -52, -12, -12 |
| Superior temporal sulcus | 125 | 4.52 | -52, -40, 4 |
| Anterior temporal lobe | 53 | 4.43 | -54, 8, -22 |
| Orbitofrontal cortex | 52 | 4.40 | 30, 52, -14 |
| Precentral gyrus | 90 | 4.31 | -38, -16, 56 |
| Supramarginal gyrus | 54 | 4.28 | 48, -36, 54 |
| Fusiform gyrus | 54 | 4.11 | -26, -66, -10 |
| Supramarginal gyrus | 75 | 4.09 | 60, -26, 46 |
| IFC pars orbitalis | 47 | 4.05 | -40, 30, -12 |
| Middle frontal gyrus | 94 | 4.01 | 22, 54, 26 |
| Supramarginal gyrus | 94 | 3.95 | 62, -22, 26 |
| Precentral gyrus | 38 | 3.95 | -46, -2, 52 |

**Table S5**. Results from a two-way ANOVA with factors valence (desirable and undesirable) and character (in-group, mild out-group, moderate out-group and extreme out-group).

**
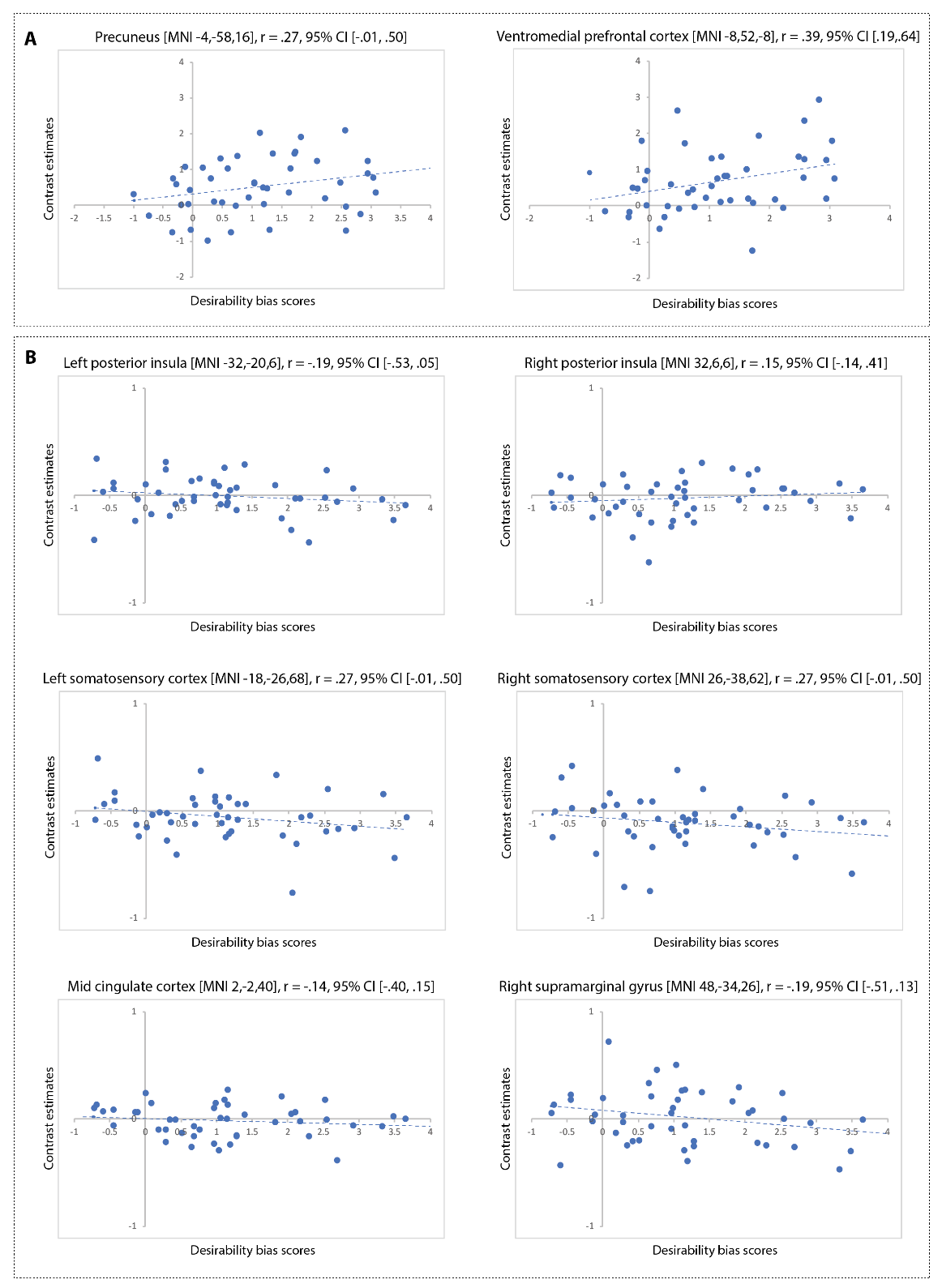
**

**Figure S1**. Scatterplots of the brain-behaviour correlations for the regions of interest following the three-way conjunction analysis (in-group - mild out-group) ∩ (in-group - moderate out-group) ∩ (in-group - extreme out-group) (**box A**) and the pairwise comparison 2 *(mild out-group - in-group) – [(moderate out-group – in-group) + (extreme out-group – in-group)] (**box** **B**).

**
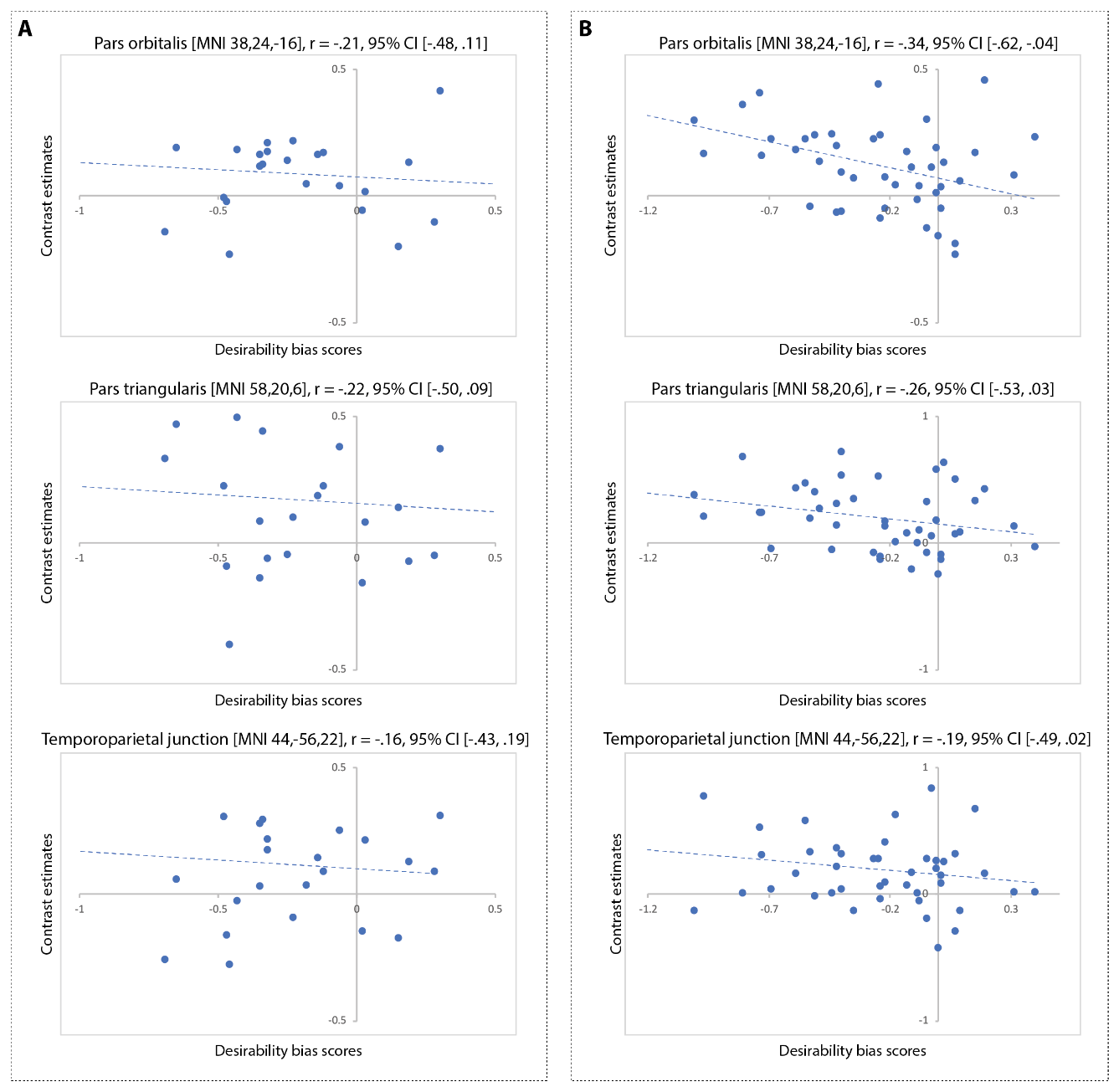
**

**Figure S2**. Scatterplots of the brain-behaviour correlations for the regions of interest following the two-way conjunction analysis (extreme out-group – in-group) ∩ (moderate out-group – in-group). **Box** **A** depicts the brain-behaviour correlations for the extreme out-group and **Box B** depicts the brain-behaviour correlation for the moderate out-group.

**
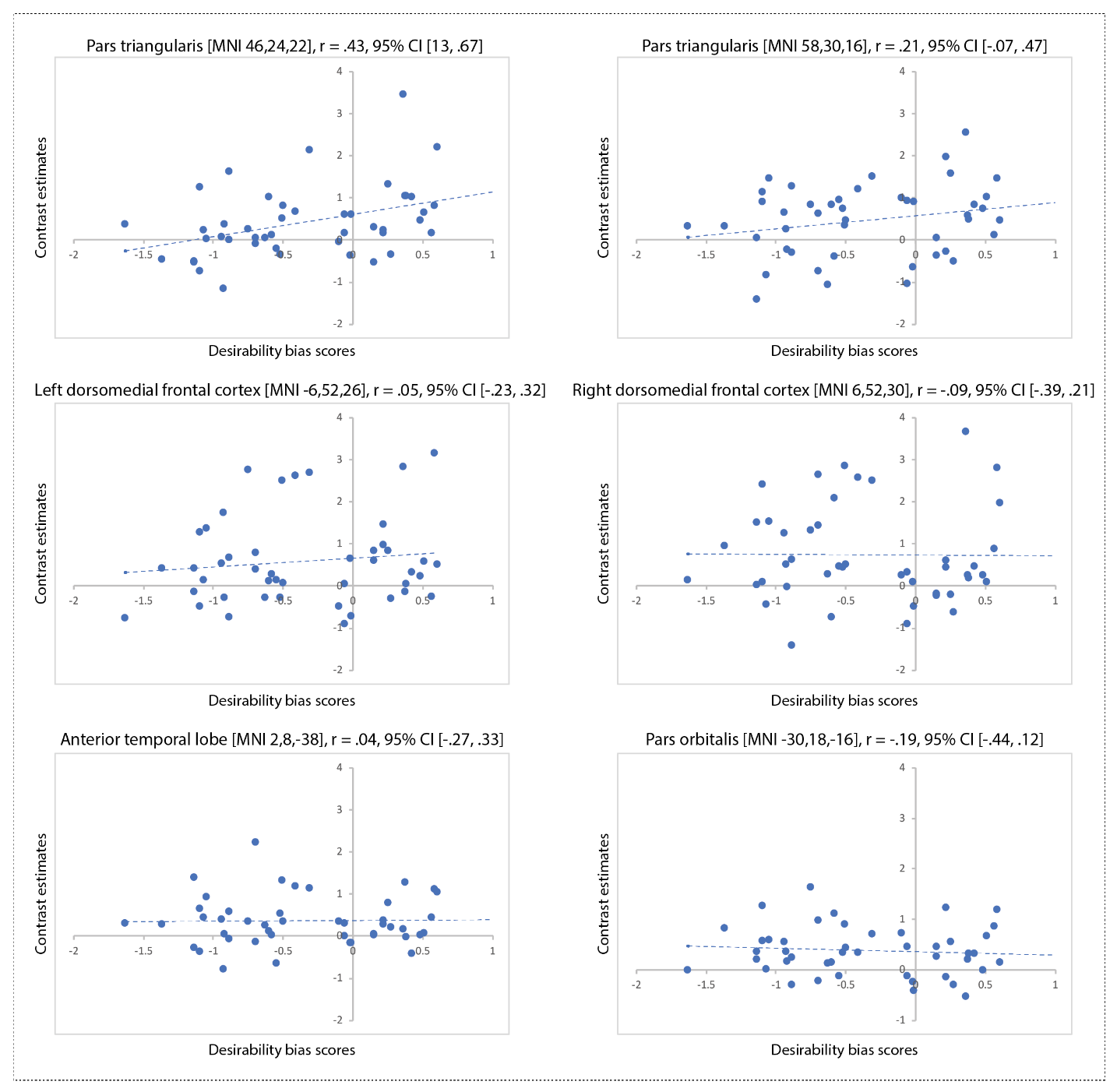
**

**Figure S3**. Scatterplots of the brain-behaviour correlations for the regions of interest following the pairwise comparison 2 *(extreme out-group – in-group) – ([moderate out-group – in-group] + [mild out-group – in-group]).

**
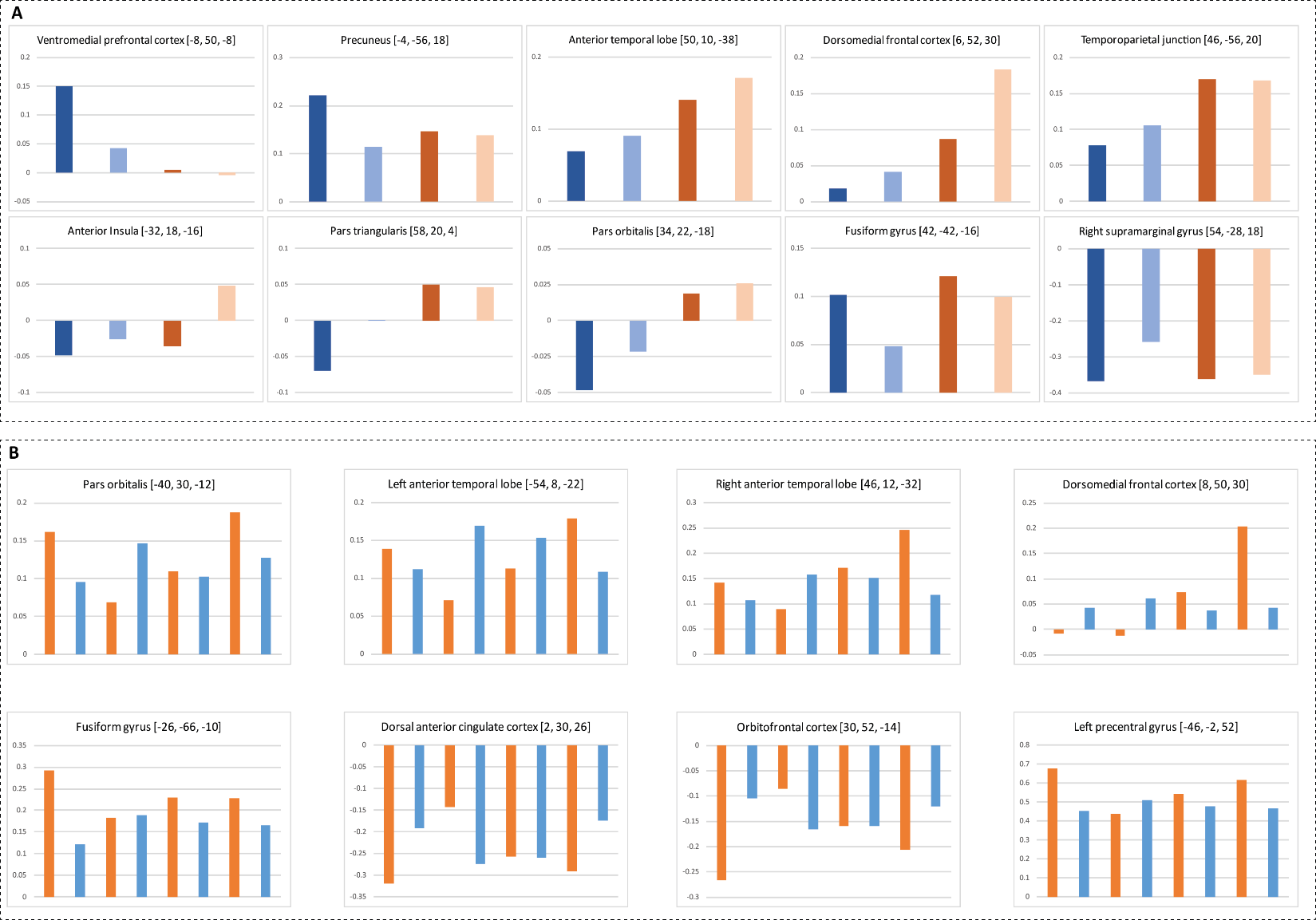
**

**Figure S4**. Contrast estimates for the most important brain regions found in the 4 (character) x 2 (valence) ANOVA. **Box A** depicts the results from the main effect of character (four levels: in-group, mild out-group, moderate out-group and extreme out-group). **Box B** depicts the results from the interaction effect character x valence.


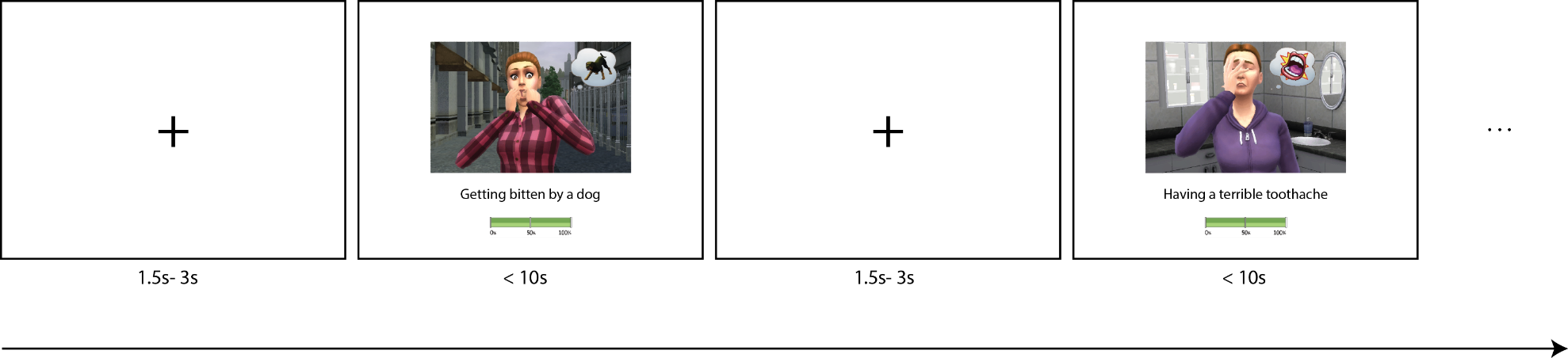


**Figure S5**. Layout of the experimental design. Following on-screen introduction of the four fictional characters and explanation of the task, participants rated the likelihood estimates of each character experiencing each of the thirty-two target events. Each of the one hundred and twenty-eight trials began with a fixation cross with a jittered duration of 1.5-3s, followed by a single screen depicting the target character in the target event, a one-sentence description of the target event and the visual analogue scale from 0% to 100%.
